# Single-cell RNA sequencing reveals different cellular states in malignant cells and the tumor microenvironment in primary and metastatic ER-positive breast cancer

**DOI:** 10.1038/s41523-025-00808-w

**Published:** 2025-08-26

**Authors:** Furkan Ozmen, Tugba Y. Ozmen, Aysegul Ors, Mahnaz Janghorban, Matthew J. Rames, Xi Li, Aaron Reid Doe, Fariba Behbod, Gordon B. Mills, Hisham Mohammed

**Affiliations:** 1 Division of Oncological Sciences, Knight Cancer Institute, Oregon Health & Science University, Portland, OR USA; 2 Cancer Early Detection Advanced Research Center, Knight Cancer Institute, Oregon Health & Science University, Portland, OR USA; 3 University of Kansas Medical Center, Kansas City, KS USA

**Keywords:** Breast cancer, Cancer genomics, Cancer microenvironment, Metastasis, Tumour angiogenesis, Tumour biomarkers, Tumour heterogeneity, Tumour immunology

## Abstract

Metastatic breast cancer remains largely incurable, and the mechanisms driving the transition from primary to metastatic breast cancer remain elusive. We analyzed the complex landscape of estrogen receptor (ER)-positive breast cancer primary and metastatic tumors using scRNA-seq data from twenty-three female patients with either primary or metastatic disease. By employing single-cell transcriptional profiling of unpaired patient samples, we sought to elucidate the genetic and molecular mechanisms underlying changes in the metastatic tumor ecosystem. We identified specific subtypes of stromal and immune cells critical to forming a pro-tumor microenvironment in metastatic lesions, including CCL2+ macrophages, exhausted cytotoxic T cells, and FOXP3+ regulatory T cells. Analysis of cell-cell communication highlights a marked decrease in tumor-immune cell interactions in metastatic tissues, likely contributing to an immunosuppressive microenvironment. In contrast, primary breast cancer samples displayed increased activation of the TNF-α signaling pathway via NF-kB, indicating a potential therapeutic target. Our study comprehensively characterizes the transcriptional landscape encompassing primary and metastatic breast cancer.

## Introduction

Breast cancer, which remains the most prevalent cancer in women, is a diverse and complex disease with a wide range of clinical manifestations and outcomes. The shift from an early localized primary tumor to metastatic lesions in distant organs represents a pivotal moment in the clinical course and prognosis of the disease. Despite advances in early detection and treatment, progression to metastatic disease remains a significant clinical challenge with an unfavorable prognosis^1^. Individuals diagnosed with localized breast cancer typically exhibit an overall survival rate exceeding 90%^2^. Conversely, the prognosis drastically declines when cancer progresses to distant metastasis, with survival rates plummeting to around 25%^3^. Therefore, understanding the complex mechanisms and differences between primary and metastatic breast cancer is essential for informing treatment approaches and improving patient outcomes.

Tumor metastasis requires a complex, orchestrated cascade involving the inherent characteristics of tumor cells, such as genetic mutations, and the intricate interplay between cancer cells and different cellular elements within the tumor microenvironment (TME). This dynamic interaction encompasses a range of participants, including immune cells, tumor-associated macrophages (TAMs), lymphoid cells, cancer-associated fibroblasts (CAFs), and components of the extracellular matrix (ECM)^4–6^. Understanding the complex interactions between different cellular components in the TME is crucial for comprehending the mechanisms of tumor initiation, progression to metastasis, and prognosis. Studies have uncovered mutational and transcriptional signatures that are more frequent in breast cancer metastases using bulk genomic sequencing methods^7,8^. However, such analyses cannot pinpoint the sources of observed differences or capture the dynamic cellular interplay between the cell types shaping the metastatic microenvironment in advanced breast cancer.

Single-cell RNA sequencing (scRNA-seq) can reveal the distinct transcriptional profiles of individual malignant and non-malignant cells in the tumor ecosystem. This has enabled analysis of complex intra-tumoral heterogeneity among TME interactions in triple-negative breast cancer^9^ as an example. In particular, previous studies have suggested that FOXP3+ regulatory T cells (Tregs) in breast cancer may lead to immune tolerance and poorer overall survival^10^, while cytotoxic T cells with exhausted gene expression patterns might characterize an immunosuppressive TME^11^. Although immune deregulation is undeniably a core component of the transition to metastatic disease, comprehensive transcriptomic profiling comparing the primary and metastatic breast cancer TME at single-cell resolution has only been applied to a limited number of cases and metastatic sites^12^.

Here, we conduct scRNA-seq analysis to deconvolve the TME landscape in primary and metastatic ER+ breast cancer. We elucidate distinct gene expression profiles of tumor cells in unpaired primary and metastatic ER+ breast cancer samples while identifying specific subtypes of stromal and immune cells that may collectively contribute to developing an immunosuppressive microenvironment within metastatic tumors. Our study provides an overview of the underlying functional landscape of primary and metastatic breast cancers while shedding light on the heterogeneity and transcriptomic TME patterns underpinning disease progression.

## Results

### Landscape of primary and metastatic breast cancer via scRNA-seq

**Fig. 1:**
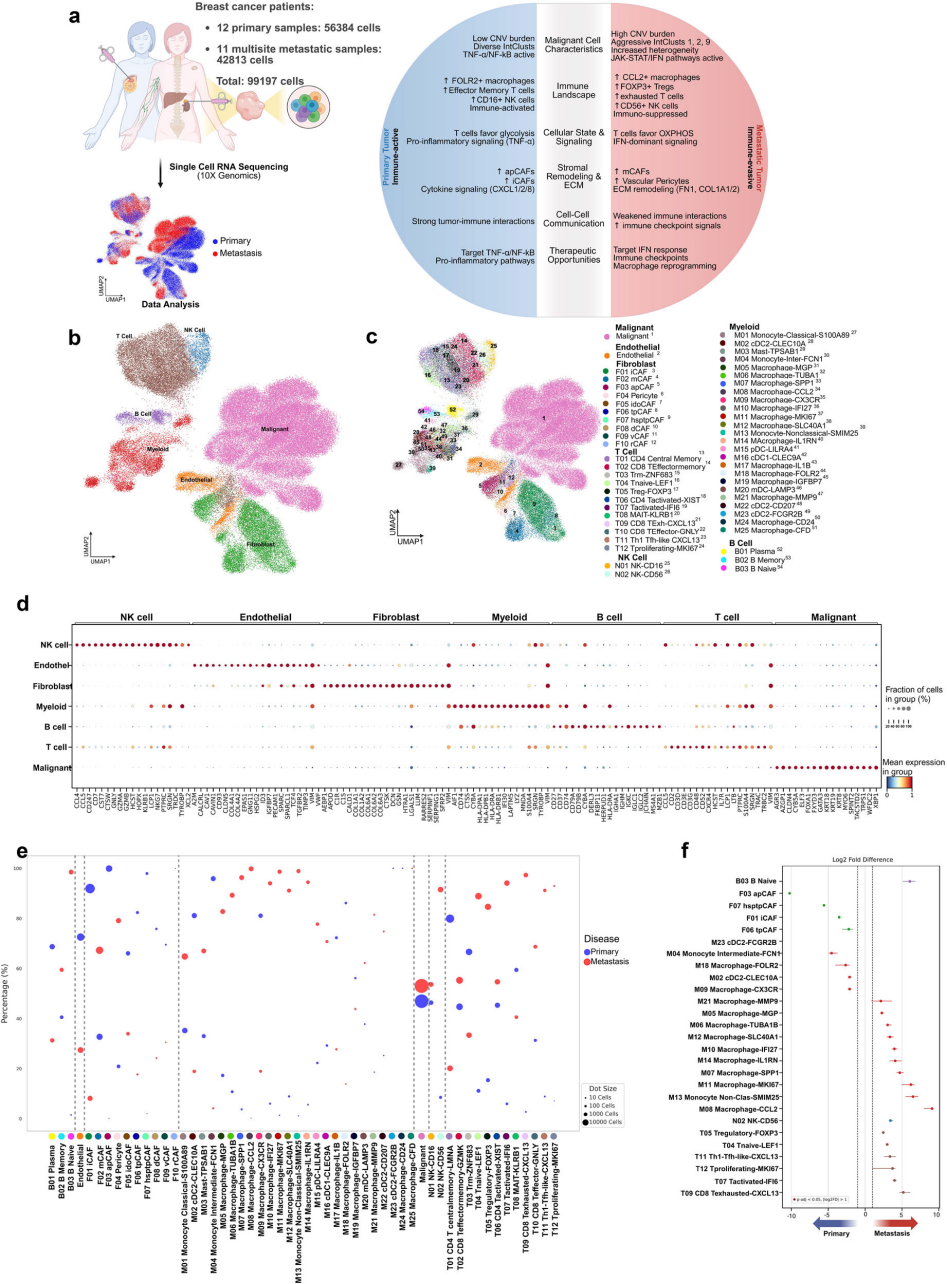
Single-cell landscape of primary and metastatic ER+ breast cancer: characterization and cellular subtype differences. **a** Sample collection workflow and data analysis overview with a summary of key findings (created using BioRender; BioRender.com/y20n356). **b** UMAP visualization of the unified cell map, showing seven major cell types colored by cluster. **c** UMAP visualization of the same cell map, displaying 54 minor cell types uniquely colored within the seven major cell types. **d** Dot plot showing differentially expressed genes across the seven major cell type clusters. **e** Relative percentage of the 54 minor cell types stratified by metastatic status; dot size indicates the number of cells. **f** Statistically significant changes in minor cell type proportions between primary and metastatic breast cancer samples.

Despite the limited survival of patients with metastatic breast cancer, little is known about the evolution that occurs between normal and malignant cells in the tumor ecosystem of primary and metastatic breast cancer. To investigate this issue, scRNA-seq was performed on an all-female patient cohort comprising individuals diagnosed with either primary (*n* = 12) or metastatic ER+ breast cancer (n = 11). Biopsies were obtained from unpaired patients at multiple metastatic sites, including the liver, bone, lymph nodes, mediastinum, adrenal gland, and skin. (Fig.1a). All patients were classified as estrogen receptor-positive (ER+) based on IHC analysis (Supplementary Data 1).

To minimize technical variability during sample preparation, all tumor biopsies were processed using a standardized experimental protocol for tissue dissociation, single-cell suspension generation, and scRNA-seq library construction. After rigorous and uniform quality control, including mitochondrial content filtering, gene/UMI thresholds, and doublet removal, each sample was processed and analyzed using consistent parameters to ensure comparability across individuals. To further mitigate batch effects and account for inter-patient variability, we applied metadata-aware integration using SCVI^13^, incorporating biopsy identity as a covariate to model sample-specific variation. We then implemented SCANVI^13^ and CellHint^14^ for biology-aware integration, leveraging known cell type labels to improve annotation accuracy and resolution. Following principal component analysis, a total of 56,384 single cells from primary breast cancer tissues and 42,813 single cells from metastatic tissues (99,197 cells in total) were embedded in UMAP space for downstream analysis. The cells were partitioned into fifty-four clusters, consisting of seven main cell types: malignant cells, myeloid cells, T cells, natural killer (NK) cells, B cells, endothelial cells, and fibroblasts (Fig. 1b, c). Each cell type was characterized using established gene expression markers^14–16^ (Fig. 1d). Additional details and corresponding references for the methodologies employed can be found in ‘Methods’.

Copy number variation (CNV) profiles were determined using gene expression data and used to identify normal and malignant cells (see Methods). While both primary and metastatic samples exhibited the same main cell types, the proportions of each cell type varied widely between patients (Supplementary Fig. 1a, b). However, when we explored the differences in minor cell types (subtypes) within each group, we found a clear distinction in the proportion of cellular subtypes associated with primary and metastatic disease (Fig. 1e, f, Supplementary Fig. 1c). Malignant epithelial cells were present in similar proportions in both primary and metastatic samples. Primary tumor samples showed higher enrichment for FOLR2 and CXCR3 positive macrophages, which have been associated with a pro-inflammatory phenotype^17–20^. In contrast, macrophages positive for CCL2 and SPP1, which have been associated with a pro-tumorigenic subtype^21,22^, were more abundant in metastatic samples. These subtype-specific shifts indicate distinct microenvironmental remodeling events that may actively drive metastatic progression.

### Genomic and phenotypic alterations within malignant cells

**Fig. 2:**
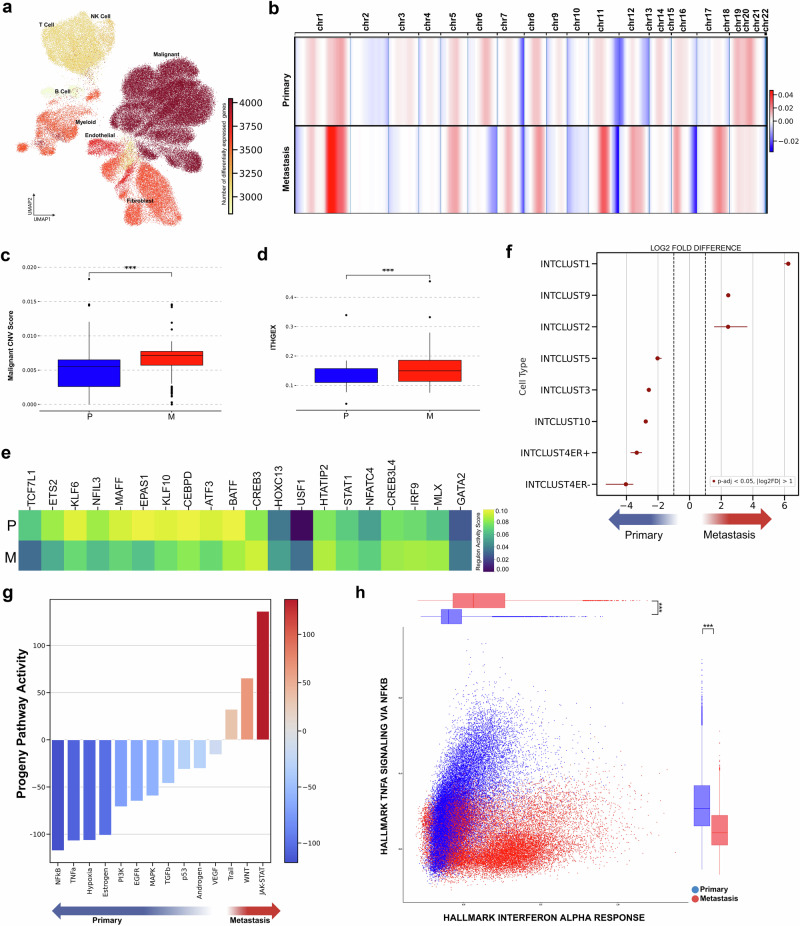
Genomic and phenotypic alterations in malignant cells of primary vs. metastatic breast cancer: insights from CNV analysis, gene regulatory networks, and integrative clusters. **a** UMAP visualization showing the number of differentially expressed genes across all major cell type clusters. **b** Chromosomal copy number variation (CNV) alteration score by metastatic status, with copy number gains and losses represented in red and blue, respectively. **c** CNV score comparison between malignant cells from primary and metastatic tumors (***p < 0.001). **d** ITGEX score comparison between malignant cells from primary and metastatic tumors (***p < 0.001). **e** Regulon activity scores in malignant cells, stratified by metastatic status. **f** Log2 fold-change analysis of integrative cluster differences between primary and metastatic malignant cells (adjusted *p* < 0.05). **g** Differential Progeny pathway activity scores in malignant cells across metastatic status. **h** Cellular states plot showing Hallmark gene signature activity for Interferon Alpha Response versus TNFα signaling via NF-κB in malignant populations.

To identify cell types that exhibit high levels of gene expression differences between the primary and metastatic sample groups, we performed differential gene expression analysis for each major lineage among patients. Results indicated that malignant cells exhibited the most remarkable diversity of differentially expressed genes (DEGs), indicating pronounced transcriptional dynamics within these cellular populations (Fig. 2a). We then aimed to characterize the tumor phenotype and clonal substructure in high detail. We inferred CNV using two independent tools: InferCNV^23^ and CaSpER^24^. T cells were used as a reference for each condition (primary/metastasis) (Fig. 2b, Supplementary Fig. 2a, Supplementary Data 4).

CNV analysis revealed substantial copy number alterations in both primary and metastatic disease, with notable inter-patient variability within each group. When comparing the overall CNV landscape between primary and metastatic tumors, we identified significant inter-site differences, particularly on chromosomes 1, 6, 11, 12, 16, and 17. Whilst the observed CNV differences may be linked to metastatic transition, it is important to note that these differences could also reflect underlying variations in patient genetics and sample composition. To address potential confounding effects arising from inter-patient variability, we compared our findings to publicly available paired primary and metastatic ER+ samples from the Pal, Chen and Valliant et al. dataset^25,26^. We employed the InferCNV algorithm^23^ to identify and extract data from malignant cells from each dataset for a more precise assessment of the CNVs. While some variability was expected, the overall CNV patterns showed a reasonable degree of concordance across datasets, with notably similar alterations observed on chromosomes 1, 11, 12, 16, and 17 within metastatic malignant cells (Supplementary Fig. 3a).

To further assess the consistency of CNV changes across datasets, we grouped genes by chromosomal order and proximity using InferCNV and performed a permutation test with 10,000 iterations (*p* < 0.05) to identify significant CNV groups distinguishing primary and metastatic malignant cells. Comparison of significant CNV regions between our dataset and the external cohort revealed a 73.40% overlap, reinforcing the robustness and generalizability of our findings (see Methods; Supplementary Fig. 3b). Further, larger cohort studies would enable deeper profiling of single-cell CNV patterns, better accounting for potential confounding factors^27^.

To comprehend the distinct clonal structures between primary and metastatic breast cancer patients, we integrated CNVs from all tumor subpopulations per patient. We compared the overall pattern of copy number alterations across chromosomal arms. Our analysis led us to identify the top 25 CNVs within chromosomal arms that were specific to metastatic or primary subclones (Supplementary Fig. 2b). In particular, CNVs in specific chromosomal regions, namely, chr7q34-q36, chr2p11-q11, chr16q13-q24, chr11q21-q25, chr12q13, chr7p22, and chr1q21-q44, were more frequent in the metastatic samples. Intriguingly, these regions encompass genes that have previously been associated with progression and aggressiveness of different cancer types, including ARNT, BIRC3, EIF2AK1, EIF2AK2, FANCA, HOXC11, KIAA1549, MSH2, MSH6, and MYCN^28–35^. These genes are associated with various aspects of cancer development and progression, including cell growth, proliferation, metabolism, and survival^28–35^.

We then calculated CNV scores for each cell (see Methods) using InferCNV, which represents the extent of copy number variations within a cell and reflects genomic instability. We found higher CNV scores in tumor cells from metastatic patient samples compared to primary breast samples (Fig. 2c). This finding is consistent with previous studies that have linked high CNV scores to poor prognosis in various types of cancer^36,37^. Besides intertumoral heterogeneity, intratumoral heterogeneity presents another significant challenge in accurately depicting the genomic landscape^38^. To further investigate intratumoral heterogeneity of copy number alterations, we used the SCEVAN^39^ algorithm to identify tumor sub-populations with different copy number alterations for each sample. Our observations indicate that metastatic tumors have a higher intratumoral heterogeneity gene expression score (ITHGEX)^40^ compared to primary tumors, consistent with metastatic breast cancer samples exhibiting higher levels of intratumoral heterogeneity (Fig. 2d). This is further evidenced by the existence of more tumor subclones within metastatic samples (Supplementary Fig. 2b, Supplementary Data 2).

### Gene regulatory signatures across primary and metastatic breast cancer

Next, we aimed to uncover the cellular processes potentially involved in primary and metastatic breast cancer by constructing lineage-specific gene regulatory networks (GRNs) based on transcription factor (TF) activity and their associated targets using SCENIC^41^. Our study revealed distinct expression patterns of the transcription factors associated with breast cancer pathophysiology (Fig. 2e).

Primary breast cancer malignant cells demonstrated higher regulon activities of transcription factors, including ETS2, EPAS1, BATF, NFIL3, TCF7L1, KLF6, MAFF, KLF10, CEBPD, and ATF3. These transcription factors have previously been reported to participate in processes such as tumor development, apoptosis, immune cell differentiation, energy metabolism, and regulation of essential signaling pathways^42–48^.

In metastatic breast cancer, higher regulon activities of transcription factors, including HOXC13, GATA2, IRF9, MLX, CREB3L4, NFATC4, STAT1, HTATIP2, USF1, and CREB3, which have been associated in prior studies with diverse biological processes relevant to cancer progression^49–53^.

The differential activities of specific regulons in malignant cells reveal distinct mechanisms that could potentially be targeted in primary and metastatic tumor states.

### Metastatic breast cancer displays an enrichment for more aggressive Integrative Clusters

We classified individual cells using Integrative Clusters (IntClust) derived from the METABRIC study^54^. The classification was based on raw expression data derived from the METABRIC study, using the top 200 DEGs for each IntClust. To compute scores for each IntClust and classify each malignant cell, we employed the Cluster Independent Annotation (CIA) tool^55^. Finally, we performed a statistical proportion analysis for each cluster to determine whether any cell type was preferentially enriched or depleted in metastatic or primary samples.

Malignant cells from primary tumors showed an increased presence of signatures associated with IntClust3, 4ER+, 4ER−, 5, and 10. IntClust3 is characterized by distinct patterns of chemosensitivity, and IntClust10 is associated with a stable probability of relapse-free cases among ER patients after five years^56^. In contrast, in malignant cells from metastatic tumors, we observed significant enrichment of signatures associated with IntClust1, 2, and 9. These three IntClust types are associated with more aggressive tumor behavior^54–56^. IntClust1 is characterized by late-recurring ER-positive genomic subgroups^56^, IntClust2 is associated with genomic alterations that contribute to its aggressive behavior^56^, and IntClust9 is distinguished by amplification of the MYC oncogene at 8q24 in 89% of tumors^56^ (Fig. 2f). Furthermore, we observed significant intratumor heterogeneity in the expression of IntClust types, with varying expression profiles that encompass multiple IntClust subtypes. The dominant IntClust types, representing the most frequent cellular subtypes, varied across samples, reflecting substantial heterogeneity. (Supplementary Fig. 3c).

In addition to IntClust, we also applied the PAM50 classification to further annotate malignant cells in our data. Using transfer learning from the publicly available scRNAseq dataset by Wu, Al-Eryani, Roden et al. ^57^, we classified malignant cells into PAM50 subtypes, including Luminal A, Luminal B, HER2-enriched, Cycling, and Basal-like stem cells. Consistent with the predominance of ER-positive breast cancer (luminal subtype) in our dataset, most malignant cells were annotated as luminal (Supplementary Fig. 3d, e). However, we observed notable variability in cell-type distribution across individual samples and between primary and metastatic tumors. Similar to the IntClust analysis, each malignant tumor core contained multiple annotated cell types, though the dominant subtype differed between tumors.

### Primary breast cancer samples displayed increased activation of the TNF-α signaling pathway via NFkB

TNF-α and NFkB signaling pathway activities were elevated in malignant cells of primary breast cancer samples compared to those of metastatic samples. In contrast, we observed higher JAK-STAT pathway activity in malignant cells from metastatic samples compared to primary samples using Progeny pathway activity^58^ (Fig. 2g, h). We further investigated highly expressed genes from the NF-kB and JAK-STAT pathways in malignant cells from primary and metastatic samples. We found that many of the JAK-STAT-related genes enriched in malignant cells from metastatic tumors were also associated with interferon-related functions. In contrast, malignant cells from primary breast tumors were enriched for genes associated with chemotaxis and immune regulation, including CCL20 and CXCL2^59,60^ (Supplementary Fig. 4g, h). A similar trend was observed at the single-cell level, where malignant cells from primary samples were mostly in a cell state defined by higher TNF-α signaling via NF-KB, whereas malignant cells from metastatic samples were enriched in either hypoxia or interferon-alpha response cell states (Supplementary Fig. 4a). To validate our findings, we analyzed the TCGA BRCA dataset^30^, restricting our analysis to ER+ breast cancer samples (Supplementary Fig. 4b, c). We found that patients with high TNF-α signaling via NF-κB, as determined by Hallmark gene signatures, exhibited improved overall survival. Additionally, using paired primary and metastatic samples from the Pal, Chen and Valliant et al. dataset^25,26^, we confirmed that Hallmark IFN-gamma response, which was elevated in metastatic samples in our study, followed a similar pattern in most patients in their study (Supplementary Fig. 4d).

To further characterize the molecular features distinguishing primary and metastatic malignant cells, we defined a metastatic malignant cell gene signature by selecting the top 200 differentially expressed genes upregulated in malignant cells from metastatic tumors compared to malignant cells from primary tumors. We then assessed whether this transcriptional program was preserved in other datasets by evaluating the enrichment of our metastatic gene signature across independent datasets. In the TCGA BRCA dataset^30^, our metastatic gene signature was enriched in the poor-prognosis group, consistent with its enrichment in clinically aggressive disease (Supplementary Fig. 4e). Similarly, in the Pal, Chen and Valliant et al. dataset^26^, most patients exhibited higher expression of metastasis-specific gene signatures in their metastatic biopsies. (Supplementary Fig. 4f).

Given the importance of these pathways in regulating the immune response and reshaping the tumor microenvironment, we further investigated changes within non-malignant cells to assess potential deregulations in the immune microenvironment.

### Macrophage subtypes are polarized toward immunosuppression across metastatic breast cancer

**Fig. 3:**
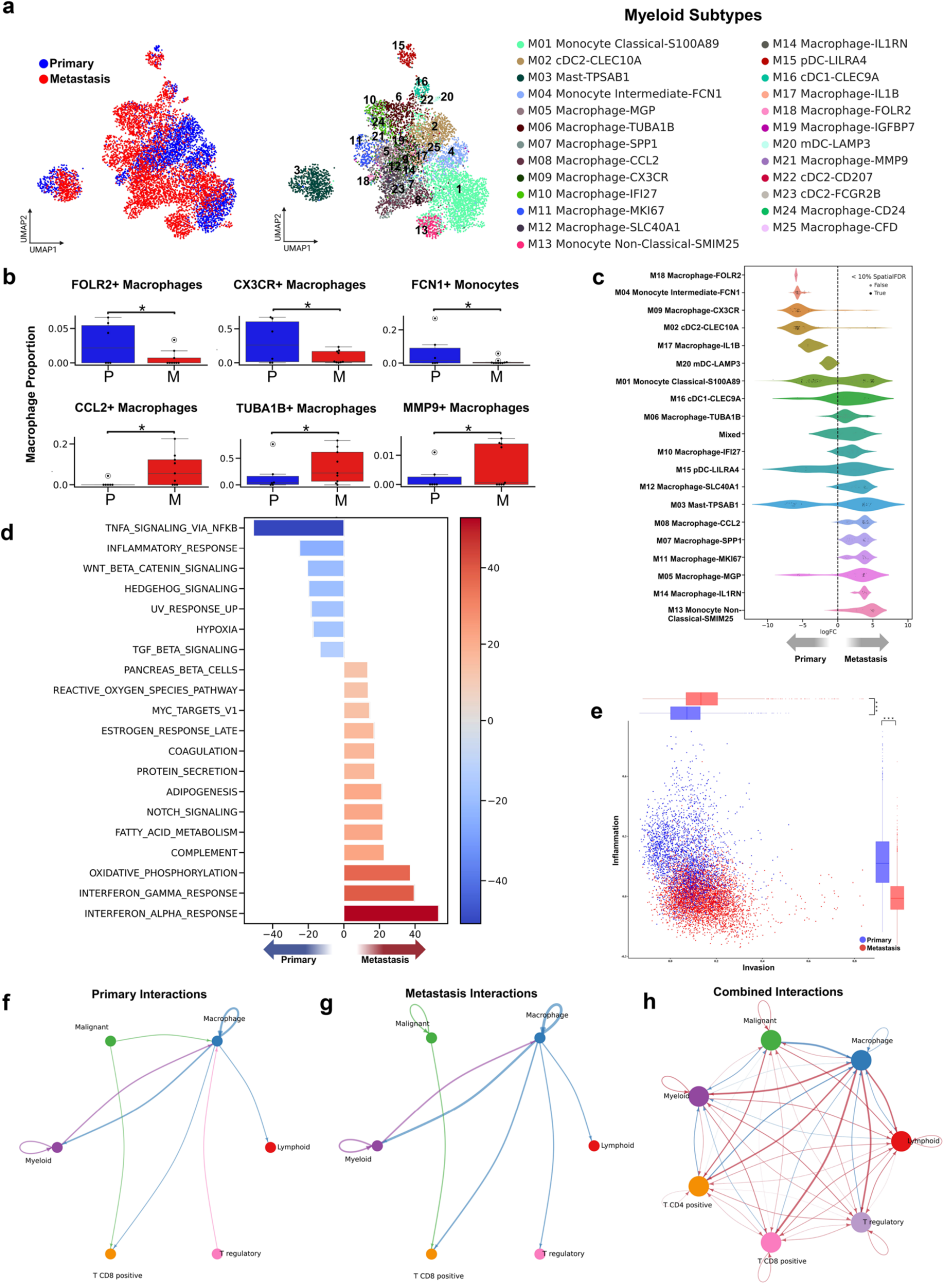
Distinct myeloid cell populations and their roles in primary and metastatic breast cancer: insights into macrophage polarization and tumor microenvironment interactions. **a** UMAP visualizations of 7,651 myeloid cells, colored by tissue class of origin (left) and clustered into 25 subtypes (right), including six dendritic, three monocytic, one mast, and fifteen macrophage lineages. **b** Bar plots showing the proportions of cells from each tissue class (*P* = Primary, M = Metastatic) for macrophage subtypes with significant proportion differences (permutation test, *p* < 0.05, log2FC > 2). **c** Beeswarm plot illustrating differential abundance analysis of myeloid subtypes across neighborhoods, stratified by tissue class. **d** Differential Hallmark gene signature pathway activity scores in malignant cells by metastatic status. **e** Cellular states plot showing CANCERSEA signature activity for Inflammation versus Invasion in myeloid populations. **f** Circle plot depicting the top 15% of tumor microenvironment interactions in primary tumors; arrow color indicates sender and thickness represents interaction strength. **g** Circle plot of the top 15% of tumor microenvironment interactions in metastatic tumors. **h** Comparative circle plot visualizing differential cell-cell interactions; red edges indicate increased interactions in metastatic tumors, and blue edges indicate higher interactions in primary tumors.

To characterize the diversity of myeloid populations within the tumor microenvironment, we identified twenty-five distinct myeloid subclusters in both primary and metastatic cancer types, including fifteen subtypes of macrophages, six subtypes of dendritic cells (DCs), three subtypes of monocytes, and a single subtype of mast cells. Among these, specific myeloid cell subtypes such as FOLR2-expressing macrophages, CX3CR-expressing macrophages, and FCN1-expressing monocytes were notably enriched with statistical significance in primary tumors (Figs. 3b, c). These subtypes, previously defined as immune-active, have also been implicated in T cell communication and broader immune orchestration in prior studies^17,19,20,61–63^.

In the metastatic tumors, our study predominantly found a significant increase in macrophages expressing CCL2, MGP, SPP1, and MMP9, which have been previously associated with tumor cell invasion, metastasis, and immunoresistance^21,63–68^.

Using pseudobulk differential gene expression analysis, we observed a distinct partitioning of marker enrichment across myeloid cell populations exemplified by a volcano plot representation of differentially expressed genes. Top-ranking genes associated with metastasis were predominantly associated with interferon (IFN) response pathways, a pattern consistent with prior reports of chronic IFN signaling contributing to an immunosuppressive immune contexture^69^. Conversely, the genes most prominently expressed in primary tumors were associated with TNF-α/NF-kB signaling pathway, reflecting distinct transcriptional programs between primary and metastatic myeloid cell populations (Supplementary Fig. 5a).

To further characterize the functional states of myeloid cells, we analyzed hallmark pathway activity profiles across primary and metastatic tumor samples. Our analysis revealed a significant increase in TNF-α signaling through the NFkB pathway in myeloid cells from primary tumors (Fig. 3d). In contrast, myeloid cells from metastatic tumors displayed pathways associated with oxidative phosphorylation, as well as responses to IFN-alpha and gamma. These findings reflect functional diversity among myeloid cell states, as shown by pathway activity patterns observed at the single-cell level (Fig. 3d, Supplementary Fig. 5b).

Cancer-related pathway activity analysis using the CancerSEA^70^ database showed that macrophages in metastatic breast cancer exhibited traits associated with increased invasion and metastasis =. In contrast, myeloid cells within the primary breast cancer displayed increased inflammatory activity, particularly within the macrophage subset, a pattern associated with an immune-active tumor microenvironment that differs from the profiles observed in metastatic tumors (Fig. 3e, Supplementary Fig. 5c).

To study functional mechanisms in the immune microenvironment, we identified alterations in cell-cell communication and signaling interactions within the TME using CellChat^71^ (Fig. 3f–h). When comparing primary and metastatic datasets, we noticed unique patterns of cell-cell communication. In the primary dataset, there was a greater frequency of signaling from malignant cells, Treg cells, CD4+ T cells, and CD8+T cells to macrophages, indicating directional interactions between these cell types in the primary tumor context. However, in the metastatic dataset, we observed a change in this communication pattern with an increase in signaling from macrophages to Treg cells and CD8 + T cells. These shifts in communication patterns highlight differences in immune cell interaction dynamics between primary and metastatic tumor contexts. (Fig. 3f–h).

Additionally, we observed an enrichment of ligands that potentially mediate cellular interactions in both primary and metastatic cancers. We noted elevated pro-inflammatory activity interactions, specifically with CXCL1, CXCL2, and CXCL8 in primary breast cancer. In the case of metastatic breast cancer, our study revealed aberrant interactions involving FN1, COL1A1, COL1A2, COL6A1, and THY1. These ligands are involved in the formation and regulation of the extracellular matrix (ECM), a key component of the tumor microenvironment. Prior studies have shown that aberrant interactions involving these proteins can alter ECM structure and have been associated with cancer progression and metastasis^72,73^ (Supplementary Fig. 5d, e).

### Aberrant lymphoid response within metastatic breast cancer TME

**Fig. 4:**
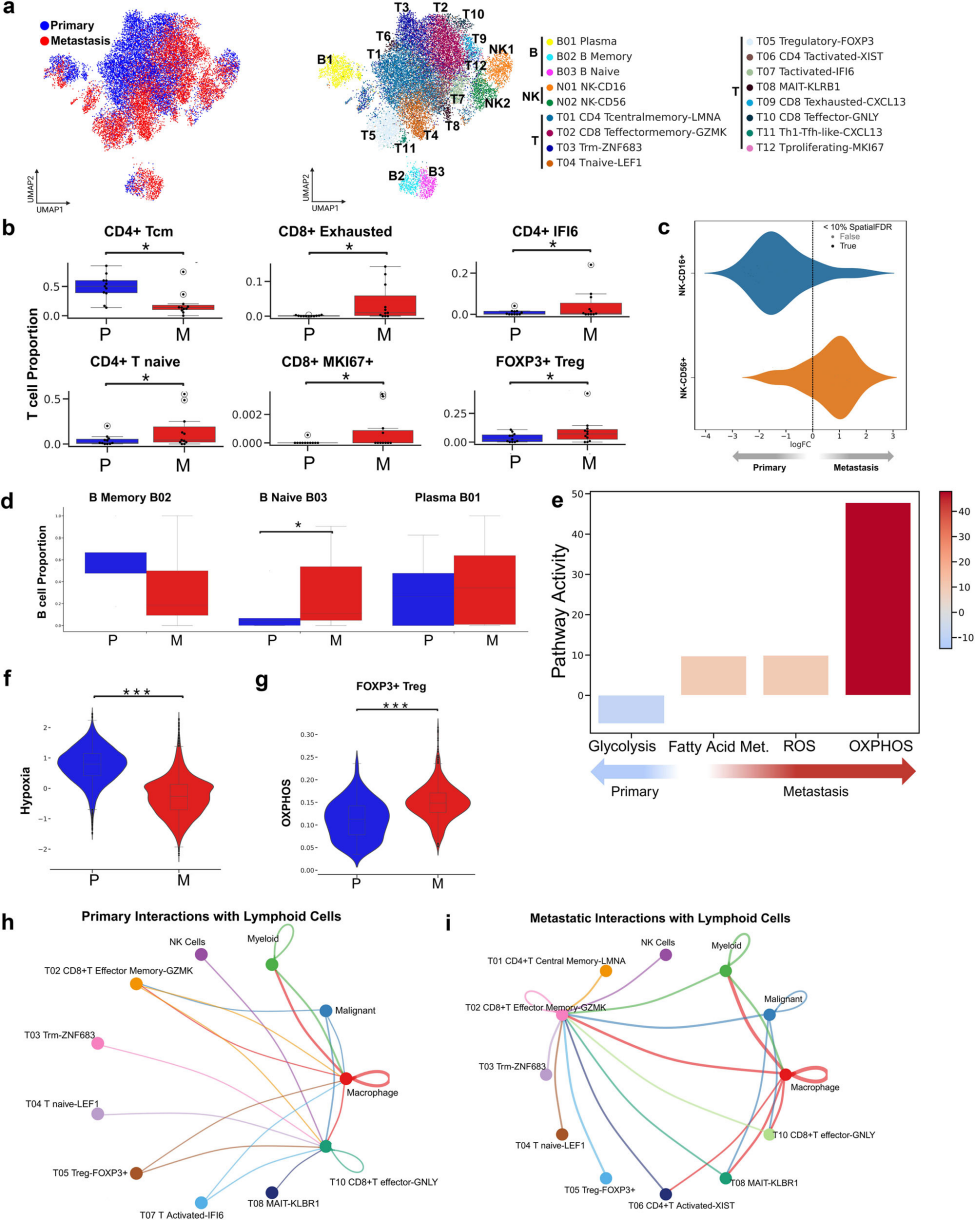
Lymphoid Cell Landscape and Functional Shifts in Primary vs. Metastatic Breast Cancer Tumors. **a** UMAP visualizations of 19,104 lymphoid cells, colored by tissue class (left) and grouped into 17 subtypes (right), including three B cell, two NK cell, and twelve T cell subtypes. **b** Bar plots showing the proportions of cells from each tissue class for T cell subtypes with significant differences (permutation test, p < 0.05, log2FC > 2). **c** Beeswarm plot illustrating the abundance differences among NK cell subtypes, stratified by tissue class. **d** Bar plots showing proportions of B cell subtypes with significant differences between primary and metastatic tissues (permutation test, *p* < 0.05, log2FC > 2). **e** Differential Hallmark gene signature activity scores within T cells across metastatic status. **f** Hypoxia score comparison between T cells from primary and metastatic tumors (***p < 0.001). **g** OXPHOS score comparison between T cells from primary and metastatic tumors (***p < 0.001). **h** Circle plot showing the top 10% of predominant interactions in the primary tumor microenvironment; arrow color indicates sender, and thickness represents interaction strength. **i** Circle plot showing the top 10% of predominant interactions in the metastatic tumor microenvironment.

To expand upon our prior findings, which highlighted the interactions between immune cells and the TME, we aimed to identify possible alterations in lymphoid subtypes during breast cancer metastasis. Unsupervised clustering of lymphoid cells identified twelve T cell, three B cell, and two NK cell subclusters (Fig. 4a) that clearly distinguished the subtypes in both primary and metastatic tumors.

Although all T-cell subtypes were present in both primary and metastatic samples (Supp. Fig. 6a), the relative proportions of certain T-cell subtypes differed significantly between primary and metastatic tumors (Fig. 4b). Primary tumors displayed a higher proportion of pro-inflammatory T-cell subtypes, including CD4+ central memory T-cells, a subtype previously associated with the expansion of antigen-specific CD4+ T-cells and promote inflammatory responses^74^. In contrast, metastatic samples exhibited mixed deregulation of T-cell subtypes, characterized by increases in CD8+ exhausted T-cells, CD4 + IFI6 T-cells, CD4+ Naive T-cells, CD8 + MKI67+ T-cells, and FOXP3+ immune regulatory Tregs (Fig. 4b). The IFI6-expressing activated T cell (Tact) subtype has been linked to the regulation of mitochondrial reactive oxygen species (ROS) and associated with disease progression and poor prognosis in TNBC and other breast cancer subtypes^22^. Similarly, the MKI67+ proliferative T cell subset has been previously observed in TNBC patients with post-chemotherapy disease progression and is associated with aggressive clinical features^22^.

Among NK cell populations, we observed a higher proportion of CD16 + NK cells in primary tumors, whereas CD56 + NK cells were more frequent in metastatic tumors (Fig. 4c). CD16-positive NK cells contribute to antibody-dependent cellular cytotoxicity (ADCC), a process where specialized immune cells, such as NK cells, recognize and eliminate cells coated with antibodies through interaction with their CD16 receptors^75,76^. Additionally, our data revealed that primary tumors show significant enrichment in ADCC-related gene signatures compared to metastatic samples as published previously^77^ (Supp. Fig. 6b).

Regarding B-cell subtypes, we found a decrease in memory B cells and an increase in naive B-cells within the metastatic samples (Fig. 4d). This pattern is consistent with the presence of B cells in the tumor microenvironment that have not undergone prior priming against malignant cells.

### Lymphoid metabolic reprogramming contributes to an immunosuppressive metastatic TME

To further probe lymphoid subtypes, we performed pathway enrichment and interaction analyses to decipher phenotypic changes. When considering all types of lymphoid cells, T cells in primary tumors show a preference for glycolytic signaling, while T cells in metastatic tumors are more enriched for fatty acid metabolism, reactive oxygen species production, and oxidative phosphorylation (Fig. 4e).

While our data shows higher hypoxic signaling in metastatic malignant cells, in primary samples, we rather observe this enrichment in the non-epithelial cell types. Indeed, we see a collective increase in hypoxic signaling, along with a decrease in oxidative phosphorylation in T cells from primary tumors compared to T cells from metastatic tumors (Fig. 4f, g). Previous studies have shown that low oxygen tension in T cells can be associated with both functional inhibition and enhancement of certain adaptive immune responses^78^. Hypoxia during antigen recognition has also been reported to influence T cell priming in ways that may support antitumor activity^79,80^.

Network analysis revealed a dramatic shift in lymphoid cell-related signaling in metastatic tumors (Fig. 4h, i). Malignant and lymphoid interactions in primary tumors were dominated by an immunostimulatory regime, which largely signaled CD8 + T effector cells, while metastatic interactions were dominated by signaling into CD8 + T effector memory (TEM) cells. The loss of the CD8+ cytotoxic population with a concomitant increase in CD8 + TEM is consistent with a reorganization of the immune landscape in metastatic lesions. CD8+ TEMs have been shown to retain antigen recognition capacity but exhibit reduced cytotoxic function under chronic stimulation in the tumor microenvironment^81,82^.

Our study observed varying levels of mediators of immune checkpoint inhibition across different cell types in both primary and metastatic breast cancers. Specifically, Tregs and Th1-like CXCL13 + CD4+ T cells showed elevated expression of transcripts for immune checkpoint mediators. Also, metastatic lesions displayed a stronger immune checkpoint inhibition signal than primary tumors (Supp. Fig. 6c).

Th1-like CXCL13^+^ CD4^+^ T cells, which have been reported to interact with regulatory T cells (Tregs) and downregulate genes involved in TCR signaling^83^, were found to be more prevalent in metastatic samples, along with Tregs (Supplementary Fig. 6a).

### Stromal cell remodeling occurs in the TME in metastatic sites

**Fig. 5:**
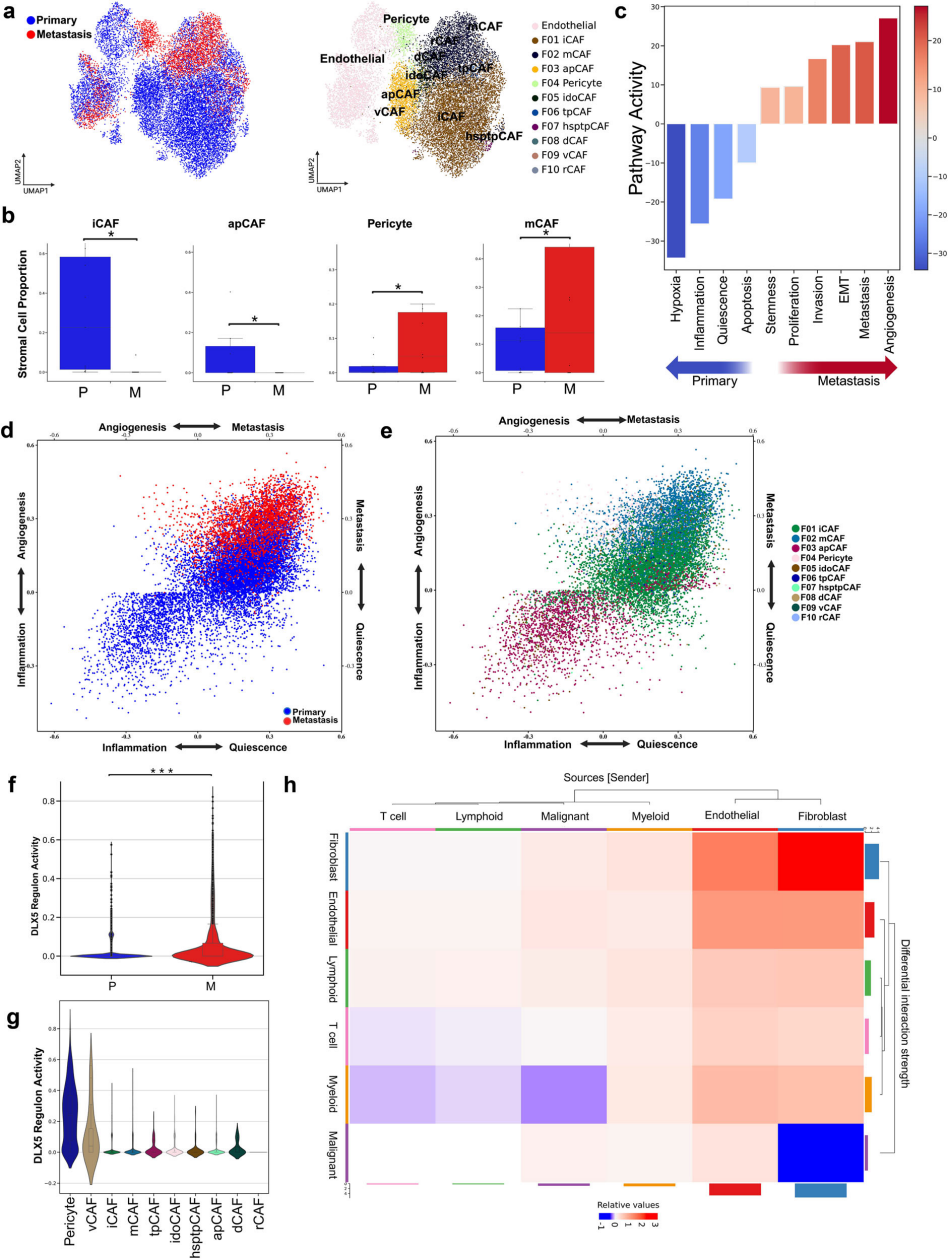
Stromal cell remodeling and fibroblast subtype dynamics in metastatic vs. primary breast cancer tumor microenvironment. **a** UMAP visualizations of 17,339 stromal cells, colored by tissue class (left) and grouped into 11 subtypes (right), including 10 fibroblast subtypes and one endothelial cell subtype. **b** Bar plots showing fibroblast subtypes with significantly different proportions between primary and metastatic tissues (permutation test, *p* < 0.05, log2FC > 2). **c** Differential CANCERSEA pathway activity scores in fibroblast cells across metastatic status. **d** Cellular states plot showing CANCERSEA gene signature activity for Angiogenesis, Metastasis, Inflammation, and Quiescence by metastatic status. **e** Cellular states plot showing the same signatures across individual fibroblast subtypes. **f** DLX5 regulon activity score comparison between stromal cells from primary and metastatic tumors (***p < 0.001). **g** Additional DLX5 regulon activity comparison highlighting consistent significance (***p < 0.001). **h** Heatmap comparing number and strength of interactions between tumor microenvironments. The top bar plot shows incoming signaling and the right bar plot shows outgoing signaling. Red indicates increases in metastatic tumors; blue indicates increases in primary tumors. Bar height reflects magnitude of change.

As key modulators of the stromal landscape of TMEs, we then focused on characterizing fibroblast subpopulations. Unsupervised clustering of stromal cells revealed changes between primary and metastatic samples across one endothelial and 10 CAF subtypes (Fig. 5a) with a loss of both antigen-presenting (apCAF) and inflammatory CAFs (iCAF) coupled with a gain of both matrix CAFs(mCAF) and pericytes (Fig. 5a, b, Supplementary Fig. 7a) in metastatic tumors.

Although CAFs are generally associated with immunosuppressive functions^84^, apCAFs have been reported to enhance antigen presentation in the tumor microenvironment through MHC class 2 expression^85^. In primary breast cancer samples, there were higher levels of iCAFs, which were identified by high expression of cytokines such as CXCL12, CXCL14, and IL6^86^. These cytokines are markers of inflammation and have been linked to signaling pathways involving PDGF, STAT3, KRAS, and complement activation^86^.

In metastatic tumors, however, we observed an increase in mCAFs, which expressed genes linked to invasive behavior, including matrix metalloproteinases (MMPs), consistent with remodeling of the metastatic breast cancer TME^86^. Vascular pericytes were also enriched in metastatic samples, in line with their previously reported involvement in cancer invasion and metastasis^87^.

As seen with myeloid and lymphoid lineages, stromal cells similarly exhibited higher levels of hypoxic signaling within the primary TME (Fig. 5c), supporting the notion that hypoxic stress shifts from non-tumor cells to tumor cells in the metastatic sites. Our findings indicate that hypoxic signaling varies between these sites, reflecting differing responses to extracellular oxygen levels rather than uniformity across cell types^88^.

Metastatic stromal cells displayed a notable increase in genes associated with angiogenesis and metastasis, indicating their potential involvement in promoting the growth and spread of tumors (Fig. 5c–e). In contrast, fibroblasts from primary TME showed an increase in inflammation and quiescence-related gene signatures.

Additionally, primary breast cancer fibroblasts displayed distinct cellular states at a cellular level, with subtypes including iCAFs within the primary TME predominantly exhibiting a quiescent state (Fig. 5d, e).

Next, we investigated the impact of transcription factors on fibroblasts. We observed high regulon activity of DLX5, a member of the MMP family^89^, especially in vascular cancer-associated fibroblasts (Pericytes and vascular CAFs) (Fig. 5f). Notably, we observed significantly higher DLX5 regulon activity in metastatic samples compared to primary tumors (Fig. 5g).

We evaluated communication between stromal cells and other cell types using the CellChat framework (Fig. 5h, Supplementary Fig. 7d). Specifically, we found that myeloid cells exhibited heightened intercellular communication with fibroblasts in primary tumors, which aligns with our analysis of communication between malignant and tumor cells. Additionally, we discovered that fibroblasts established communication channels with other cell types and within themselves, particularly in the metastatic TME.

To gain a deeper understanding of the signaling pathways influencing cell-cell interactions in the TME, we conducted network analysis utilizing the MultiNicheNet^90^ approach. Our analysis revealed significant integrin and collagen-mediated interactions among stromal cells, as depicted in (Supp. Fig. 7b, c). Integrins are known to play a critical role in cell-cell and cell-extracellular matrix interactions, while collagen is a major component of the extracellular matrix^91^. The presence of these interactions underscores the importance of integrin and collagen-mediated signaling in the communication and behavior of stromal cells within the TME, particularly in metastatic tumors^92^.

## Discussion

As primary breast cancers progress and transition to metastatic breast cancer, shifts in TME composition and signaling occur and ultimately lead to poorer outcomes^93^. The current literature has increasingly focused on understanding the complex interactions within the TME, highlighting the necessity for advanced techniques, such as scRNA-seq, to delineate the intricate cellular dynamics at play^25,94,95^.

Recent studies utilizing scRNA-seq have provided insights into the alterations occurring among cellular populations within the TME across various cancer types. While previous studies have examined metastatic breast cancer, they have been restricted to the analysis of a limited number of lymph node metastases^12,96^. In this study, we analyzed primary ER+ breast cancers and metastases across multiple sites, including lymph nodes, liver, bone, adrenal gland, and subcutaneous (Fig. 1a), to provide a broader view of TME alterations. By focusing on luminal subtype samples, we aimed to ensure comparability while capturing the molecular and cellular dynamics underlying metastasis in this tumor lineage.

Although our differential gene expression analysis on unpaired samples revealed shared core molecular features across ER+ metastatic tumor tissues from diverse biopsy locations, distinct site-specific transcriptional patterns also emerged. Malignant cells from different metastatic sites exhibit unique gene expression signatures that likely reflect the local microenvironment and potentially specialized adaptations. However, most of the key transcriptional programs identified were broadly consistent across metastatic sites, suggesting common pathways underlying metastatic colonization and growth. These findings underscore the complexity and heterogeneity of the metastatic tumor microenvironment, highlighting the importance of considering both shared and site-specific factors when developing targeted therapies. Further studies with larger cohorts and paired samples will be essential to delineate the functional consequences of these differences and their impact on clinical outcomes. While paired primary-metastatic data would provide a more direct assessment of tumor evolution, analyzing multiple unpaired samples helps mitigate selection bias and improves the generalizability of our findings. However, the lack of paired samples and the presence of inter-tumor heterogeneity should be considered when interpreting these findings, as they may influence the observed changes attributed to primary and metastatic sites. To address this limitation, we applied a standardized experimental pipeline and used metadata-aware and biology-aware integration methods to account for batch effects and inter-patient variability. Additionally, normalization and quality control followed best practices across all samples, helping to ensure that observed transcriptional differences reflect biological variation rather than technical noise.

Despite consistent representation of major cell types across primary and metastatic tumors, notable shifts emerged in the composition of cellular subtypes, states, and interaction networks. This intra-tumoral heterogeneity underscores the complexity of metastatic progression and highlights potential avenues for targeted therapeutic strategies. Differences between primary and metastatic subpopulations may reflect adaptive mechanisms that drive treatment resistance or facilitate tumor colonization at distant sites^97^.

Malignant cells exhibited substantial transcriptional diversity, with copy number variation (CNV) analyses revealing patient-specific genomic alterations in both primary and metastatic sites. Certain chromosomal regions were recurrently altered in metastatic lesions, implicating loci previously associated with breast cancer progression and aggressiveness^28–35^. Moreover, metastatic tumors demonstrated elevated CNV scores and greater intratumoral heterogeneity, indicative of increased genomic instability and clonal diversification. Such features are hallmarks of cancer evolution and may enhance tumor plasticity, promote immune evasion, and facilitate adaptation to distant microenvironments or therapy^98^. The higher clonal diversity observed in metastatic tumors aligns with prior studies^98^, supporting the notion that metastatic dissemination is accompanied by increased evolutionary complexity, cellular fitness, and selective pressure. These findings underscore the role of genomic reprogramming in shaping metastatic behavior and highlight CNV burden and subclonal heterogeneity as potential biomarkers of disease aggressiveness.

Integrative Cluster (IntClust) classification further emphasized this shift toward a more aggressive phenotype in metastases. Enrichment of IntClust1, 2, and 9 subtypes linked to late recurrence, MYC amplification, and enhanced genomic instability further supports the notion that metastatic ER-positive breast cancer exhibits more aggressive tumor behavior compared to primary disease^54^. Notably, the presence of multiple IntClust signatures within individual tumors at single-cell resolution challenges the traditional view of molecular subtypes as static or mutually exclusive. Instead, these findings point to a continuum of dynamic transcriptional states that may evolve in response to selective pressures within the tumor microenvironment. This plasticity underscores the limitations of relying solely on genomic alterations for subtype classification, as they fail to fully capture the influence of gene expression dynamics and intercellular interactions.

Building on the concept of transcriptional plasticity, the gene regulatory architecture observed in this study reveals distinct programs between primary and metastatic tumors, reflecting context-specific adaptations to local and systemic pressures. In primary tumors, several transcription factors showed increased activity, consistent with roles previously described in the literature. ETS2 has been linked to both tumor initiation and apoptosis, suggesting a dual role in growth regulation^42^, while EPAS1 facilitates adaptation to hypoxia, a common feature of the primary tumor niche^43^. BATF and NFIL3 contribute to immune differentiation and metabolic regulation^44–46^, while TCF7L1 and KLF6 are involved in Wnt signaling and oncogenic processes^47,48^, indicating a regulatory environment that integrates immune and metabolic control.

In contrast, metastatic tumors displayed elevated activity of transcription factors associated with aggressive phenotypes. HOXC13 and GATA2 have been linked to a poor prognosis and aggressive phenotypes in a number of cancer lineages^49,50^. IRF9, an interferon response marker, is primarily known for its role in anti-viral immunity and has been linked to tumor growth and metastasis^51^. MLX coordinates lipid storage with metabolic gene expression regulation and is linked to poor prognosis^52^, whereas CREB3L4 is involved in unfolded protein response and has been associated with breast carcinoma progression^53^. These shifts, supported by prior studies, highlight the regulatory flexibility that may facilitate metastatic adaptation and survival.

Together, these findings emphasize the need for a more nuanced interpretation of molecular subtypes, one that integrates transcriptomic heterogeneity, regulatory plasticity, and microenvironmental context. A deeper exploration of these dynamic transcriptional states may reveal novel vulnerabilities that emerge during metastatic evolution and inform more effective therapeutic strategies.

The divergence in signaling pathway activity between primary and metastatic malignant cells provides further insight into the evolving tumor ecosystem. Primary tumors showed increased TNF-α/NF-κB activity, which may facilitate immune cell recruitment and local inflammation^99^. Notably, the prominence of inflammatory signaling in primary tumors implies a potential role in initiating key processes conducive to tumor progression, including tumor cell invasion and migration^100^. Specifically, this aberrant signaling may serve as an early driver in the pathogenesis of primary tumors, setting the stage for subsequent metastatic dissemination^101^. The observed loss of NFkB signaling in metastatic malignant cells may contribute to the establishment of an immunosuppressive microenvironment permissive for growth in the metastatic niche^102^. The enrichment of interferon-stimulated gene expression and the suppression of inflammatory NF-κB activity may reflect an adaptive reprogramming of malignant cells to optimize their survival and growth in metastatic niches. These signaling patterns were also observed in external datasets, including TCGA bulk RNA data from ER+ breast cancer samples^30^, as well as scRNA data from paired primary-metastatic biopsies of six female patients from Pal, Chen and Valliant et al. ^26^ supporting the broader relevance of these findings across cohorts. Together, this signaling plasticity highlights a potential mechanism through which malignant cells transition from an immune-reactive to an immune-resistant state during metastasis and points to actionable differences that may guide therapeutic targeting at distinct disease stages. However, further research is needed to fully elucidate the regulatory dynamics and functional consequences of these pathway shifts across diverse patient contexts.

Myeloid cell heterogeneity, particularly among macrophages, emerged as a key feature of the tumor microenvironment (TME) with implications for tumor progression and metastasis. In primary tumors, macrophage and monocyte subtypes associated with favorable prognosis, such as FOLR2^+^, CXCR3^+^, and FCN1^+^ populations, were enriched. FOLR2^+^ macrophages facilitate antitumor immunity by promoting chemokine-mediated crosstalk with CD8^+^ T cells, including CXCL9 and CXCR3 signaling pathways^17,19,61^. CXCR3+ macrophages, in particular, play a key role in immune activation and have been identified as promising targets in cancer. Their reduced presence in metastases suggests a shift toward an immunosuppressive microenvironment that promotes tumor progression^62^. Similarly, FCN1+ monocytes have also been associated with increased inflammatory function in other cancer types, suggesting their potential role in modulating immune responses in cancer contexts^63^.

In metastatic tumors, our study identified a significant presence of macrophages expressing CCL2, MGP, SPP1, and MMP9, which are associated with various aspects of tumor metastasis and may play a crucial role in preparing the metastatic niche for malignant cell growth^21,63–68^. CCL2-expressing macrophages have been implicated in facilitating tumor cell invasion and metastasis, supported by the critical role of the CCL2/CCR2 signaling axis in tumor-associated macrophage (TAM) development, which directly influences tumor cell survival, growth, and invasive capabilities and further high levels of CCL2 expression have been correlated with poor prognostic outcomes in cancer patients^21,64–66^. Furthermore, macrophages expressing MGP contribute to the upregulation of pro-tumorigenic factors that promote immunoresistance^67^and SPP1-expressing TAMs are linked to adverse prognoses in various cancers by potentially aiding tumor invasion through the degradation of the basement membrane via matrix metalloproteinase (MMP) expression^63^. MMP9-expressing TAMs also play a significant role in creating an environment conducive to cancer metastasis, further promoting aggressive tumor characteristics and poor patient outcomes^68^. Collectively, these findings underscore the multifaceted roles of macrophages in modulating tumor biology and highlight their potential as therapeutic targets in metastatic disease. Further studies are needed to clarify how macrophage subsets mechanistically drive metastasis.

Functional pathway analysis within myeloid cells revealed heightened TNF-α/NF-κB signaling in primary TMEs, indicative of active immune responses. In contrast, metastatic myeloid populations exhibited elevated oxidative phosphorylation and type I and II interferon signaling. Acute Type I IFN signaling response is considered a key driver for inflammation^103^. However, studies suggest that Type I IFN has a dual role in cancer and chronic inflammation. The Type I IFN response in the TME may promote pro-tumorigenic TAM infiltration in metastatic lesions^104^. However, chronic interferon stimulation has been proposed to be immunosuppressive^69^ and may contribute to the immunosuppressive TME observed in metastatic ER+ breast cancers in our study. This dynamic spectrum of myeloid cell states underscores the evolving interplay between immune activation and suppression during disease progression, with important implications for therapeutic strategies targeting the TME.

Within the lymphoid context, primary tumors exhibited a higher proportion of pro-inflammatory T-cell subtypes, while metastatic samples displayed mixed deregulation of T-cell subtypes, including significant increases in exhausted T cells, IFI6 T cells, naive T cells, MKI67+ T cells, and Tregs, suggesting a shift towards a chronic immunosuppressive tumor microenvironment. Building on this, our study highlights the potential prognostic use of specific T cell subtypes in breast cancer. The presence of IFI6-expressing activated T cells was associated with disease progression post-chemotherapy and lower metastasis-free survival rates^22^. Additionally, the presence of proliferative MKI67+ T cells was exclusive to TNBC patients experiencing disease progression after chemotherapy, and their gene expression correlated with lymph node metastases, tumor invasion, and adverse survival outcomes^22^. Our study suggests that similar processes may be involved in ER+ tumors.

Natural killer (NK) cell composition also differed, with CD16^+^ NK cells, which play a role in ADCC, being more prevalent in primary breast cancer. However, in metastatic breast cancer, there was a higher proportion of CD56-positive NK cells. This shift may suggest a loss of NK cell ADCC ability during the metastatic transition, consistent with previous studies^75,76^.

B-cell dynamics further reflect immunological remodeling: metastatic tumors displayed reduced memory B cells alongside increased naive B cells, indicative of ongoing recruitment without effective antigen priming. These patterns underscore the complexity of lymphoid-tumor interactions in metastatic progression.

Metabolic profiling revealed distinct reprogramming of lymphoid cells between tumor stages. Primary tumor T cells favored glycolytic metabolism, which may support rapid proliferation and effector functions^105^, while metastatic T cells exhibited enhanced fatty acid oxidation, reactive oxygen species generation, and oxidative phosphorylation^106^. This shift reflects the changing metabolic demands as T cells adapt to the tumor microenvironment (TME) and influences their functional capacities, potentially leading to T cell persistence and exhaustion due to chronic stimulation in the TME^107^. While low-oxygenated conditions in the T cell microenvironment have been associated with inhibiting immune function, recent studies suggest that this characteristic can enhance aspects of the adaptive immune response and improve antitumor activity^78–80^. Further mechanistic studies will be needed to dissect how these metabolic adaptations shape T cell fate and function in the metastatic TME.

Network analysis demonstrated a dramatic shift in lymphoid cell-related signaling upon metastasis. Primary breast cancer lymphoid interactions were dominated by an immunostimulatory regime, primarily signaling CD8+ T effector cells. In contrast, metastatic interactions were dominated by substantive signaling into CD8+ T effector memory cells. This suggests a chronic immunosuppressive TME in metastatic breast cancer, where CD8+ TEMs recognize malignant cells but cannot effectively mediate tumor clearance.

Furthermore, immune checkpoint molecules were differentially expressed among T-cell subsets, with Tregs and Th1-like CXCL13^+^ CD4^+^ T cells showing elevated checkpoint signals. Metastatic tumors displayed heightened checkpoint inhibition relative to primary sites, highlighting a potentially actionable immunosuppressive axis. These observations underscore a potentially immunosuppressive microenvironment in ER^+^ metastases and align with previous studies suggesting that components of the immune landscape in these tumors may contribute to immune evasion^108^. Further investigation is warranted to assess whether targeting checkpoint pathways could modulate this axis in metastatic ER^+^ breast cancer.

Endothelial cells and cancer-associated fibroblasts (CAFs) constitute essential components of the TME, with CAFs critically shaping stromal dynamics that influence tumor progression, immune modulation, and therapeutic resistance. Our analysis revealed a marked and significant transition in fibroblast populations during metastasis, characterized by a shift from antigen-presenting CAFs (apCAFs) and inflammatory CAFs (iCAFs) in primary tumors toward matrix CAFs (mCAFs) and pericytes in metastatic breast cancer. While CAFs are broadly linked to immunosuppression, apCAFs can paradoxically enhance antigen presentation within the TME^85^. The enrichment of iCAFs in primary tumors, identified by elevated cytokine expression, implicates these cells in fostering a pro-inflammatory milieu supportive of tumor growth. Interestingly, a subset of iCAFs exhibited quiescence-associated gene signatures, suggesting specialized roles in regulating cellular dormancy and inflammatory responses in the primary niche^109^. In metastatic lesions, mCAFs demonstrated gene expression profiles enriched for matrix metalloproteinases (MMPs), consistent with their established function in promoting invasion and metastasis^86,87^. The concurrent increase in vascular pericytes further implicates these cells in metastatic progression through mechanisms involving vascular remodeling and stromal crosstalk. Additional research is needed to clarify the distinct roles and underlying processes of fibroblast and endothelial subtypes in metastatic remodeling.

We observed elevated hypoxic signaling in stromal cells within the primary TME, consistent with the increased hypoxia observed in myeloid and lymphoid lineages. Notably, hypoxic signaling levels varied across different cell types, underscoring the critical influence of oxygen availability in shaping the dynamics of the TME. This variation suggests the potential role of cell-type-specific responses to hypoxia in driving tumor progression and adaptation. As tumors metastasize, the burden of hypoxic stress appears to shift predominantly to malignant cells, reflecting their metabolic reprogramming to survive and thrive in often oxygen-deprived distant microenvironments^88^. This adaptation may enhance malignant cell plasticity, invasiveness, and resistance to therapies. Understanding these nuanced, context-dependent hypoxic responses highlights the importance of considering cell-type and site-specific hypoxia dynamics when developing therapeutic strategies aimed at disrupting tumor growth and metastatic colonization.

Moreover, our analyses indicate enhanced cellular communication focused on extracellular matrix (ECM) formation and remodeling in metastatic lesions. The intensive crosstalk between fibroblasts, immune cells, and malignant cells fosters a microenvironment that may create physical and biochemical barriers to immune infiltration. This ECM remodeling alters chemokine gradients and may negatively influence antigen presentation dynamics, further dampening effective immune surveillance.

Collectively, these changes create a suppressive TME that assists in tumor progression by limiting the cytotoxic capacity of lymphoid cells and enhancing mechanisms for immune escape. Our findings emphasize the need for further research into these interactions, as understanding the precise mechanisms of immune suppression in metastatic breast cancer could lead to innovative therapeutic strategies targeting these pathways. We acknowledge that our reliance on transcriptomic and computational inference necessitates functional validation in experimental models to confirm these observations.

One of our key findings was the high activity of the DLX5 regulon, particularly in vascular CAFs and pericytes. DLX5 regulates the expression of MMPs, which are enzymes involved in degrading the extracellular matrix and facilitating tumor cell invasion. The upregulation of DLX5 in metastatic stromal cells indicates a potential mechanism by which these cells contribute to the growth and spread of tumors. Overall, the high DLX5 regulon activity observed in vascular CAFs and pericytes in metastatic samples suggests its potential as a therapeutic target^89^. The prominent presence of integrin and collagen-mediated interactions in the metastatic stroma indicates their importance in establishing metastasis. Collectively, these findings elucidate how differential hypoxia signaling, gene expression, and intercellular communication within stromal populations converge to promote tumor progression and metastatic colonization.

Despite the comprehensive insights gained, it is important to recognize that single-cell RNA sequencing has inherent limitations, including potential biases introduced during tissue dissociation, loss of spatial context, and challenges in capturing rare or transient cell states. These factors can complicate the interpretation of tumor heterogeneity and cellular dynamics. Nevertheless, the detailed resolution afforded by scRNA-seq provides critical understanding of the cellular diversity and plasticity within metastatic tumors. Integrating these findings with spatial transcriptomics, longitudinal sampling, and functional validation will be vital to deepen mechanistic insights and refine therapeutic strategies.

In summary, the integrated analysis of malignant, immune, and stromal cell dynamics in metastatic breast cancer reveals significant shifts in cellular states across different cell populations, along with alterations in malignant cell copy number variation (CNV) profiles and regulatory network activity. Dynamic changes in cell subtypes correlate with disease progression and prognosis, while immune modulation, metabolic reprogramming, and cell-cell interactions sculpt the tumor microenvironment. These interactions and their regulatory networks reveal complex pathways that underpin tumor progression and immune evasion. These findings highlight the importance of targeting specific immune subsets, cellular interactions, and regulatory networks to enhance immunotherapies and precision treatments. Further research is essential to uncover the underlying mechanisms driving these alterations and their impact on patient outcomes.

## Methods

### Ethics declarations

This study was approved by the Institutional Review Boards (IRBs) of Oregon Health & Science University (OHSU) and the University of Kansas Medical Center. All biospecimens were collected and analyzed under IRB-approved protocols: the MMTERT observational study (Mitri 2018) (OHSU IRB #16113), which covers all metastatic samples; the Cancer Early Detection Advanced Research Center (CEDAR) study (OHSU IRB #20750), which covers primary samples Primary_1 through Primary_7; and a University of Kansas Medical Center study (KUMC IRB #11513), which covers primary samples Primary_8 through Primary_12. All procedures involving human participants were conducted in accordance with the ethical standards of OHSU and the University of Kansas Medical Center, and with the 1964 Declaration of Helsinki and its later amendments or comparable ethical standards. Written informed consent was obtained from all study participants.

### Sample collection and preparation

Participant eligibility was determined by the enrolling physician, and informed written consent was obtained from all subjects. All biopsy biospecimens used in this study were prospectively collected freshly for single-cell RNA sequencing library construction.

### Tissue dissociation

Tumor tissues were disaggregated by gentleMACS kits (Miltenyi Biotec, #130-095-929) following the manufacturer’s protocol. The lysate was resuspended and filtered through a 70-µm cell strainer (130-098-462; Miltenyi Biotec Germany). Cells were collected by centrifuging (300 × g for 7 min at 4 °C) and resuspended at 700–1200 cells/µl. Live cells were isolated by EasySep Dead Cell Removal (Annexin V) Kit (STEMCELL Technologies, #17899).

### Single-cell RNA sequencing library construction

All samples were processed using standardized protocols under consistent experimental conditions, from tissue dissociation to scRNA-seq library preparation, to minimize technical variability between individuals. Single-cell suspensions were processed according to the 10xGenomics scRNAseq sample preparation protocol (Chromium Next GEM Single Cell 3’ Kit v3.1, 10xGenomics). The entire mixed cell population was further analyzed without sorting or enrichment for specific cell subtypes. Cell suspensions were uploaded into the Chromium controller, capturing GEMs that encapsulated an estimated 5000–10,000 single cells per channel. Libraries were constructed from the amplified cDNA, and sequencing was performed on the Illumina NovaSeq 6000 platform. All steps were performed according to the manufacturer’s standard protocol.

### Processing and quality control of scRNA-seq data

To ensure high-quality data, we implemented three quality control measures on the raw gene-cell-barcode matrix for each cell: the proportion of mitochondrial genes (≤20%), unique molecular identifiers (UMIs), and gene count (ranging from 400 to 100,000 and 200 to 10,000, respectively) using Scanpy^110^. Doublets were identified and removed using the Scrublet^111^ package for each sample. Normalization of total counts per cell was performed using the *normalize_total* function in the Scanpy^110^ package in Python, followed by log-normalization with the *log1p* function. Clustering was conducted using the Leiden algorithm at a resolution of 1, as provided by Scanpy^110^.

### Alignment and raw expression matrix construction

Raw sequencing data were aligned to the GRCh38 genome reference using 10X software CellRanger (Version 6.1.2) with default parameters.

### RNA velocity analysis

For RNA velocity analysis, the spliced and unspliced reads were counted using the velocyto.py^112^ package (v0.17.17) from aligned bam files generated by CellRanger. A separate loom file was generated and used to process each sample further.

### Annotation

Marker genes for each cluster were identified using a t-test implemented in Scanpy^110^. These marker genes were then used to annotate each cluster using publicly available databases such as CellMarker^15^ and PanglaoDB^16^ To determine the cell identities. These annotations were later refined using the CellTypist^14^ package and its annotate function. The model ‘Immune_All_High.pkl’ was specified, and majority voting was enabled. Through this process, seven major cell type clusters were annotated: epithelial cells, natural killer cells, myeloid cells, T cells, B cells, fibroblasts, and endothelial cells. To distinguish malignant cells, we calculated and identified large-scale chromosomal copy number variation (CNV) by inferCNV^23^ and CaSpER^24^ tools for each sample based on transcriptomes. T cells and myeloid cells were considered reference cells; epithelial cells that had differing CNV patterns and exhibited higher CNV scores were annotated as malignant cells.

Datasets were merged across different samples using SCVI^113^ based integration, the top 4000 highly variable genes were used to train the VAE models, with each biopsy as a covariate key. After training the initial VAE model, the annotated cell types were used to build an extended model with scANVI^114^ for better integration.

After integration, NK cells, myeloid cells, T cells, B cells, and fibroblasts were further classified using the following publicly available single-cell RNA seq datasets: NK cells (GSE212890)^115^, T, myeloid, and B cells (GSE169426)^116^, and fibroblasts (GSE103322, GSE132465, GSE154778, and GSE212966)^86^. This label transfer was performed using the scArches^13^ algorithm, following the best practices described previously^117^.

To mitigate the confounding effects of inter-patient variability and batch effects across unpaired samples, we applied a two-step integration approach. First, SCVI was used to perform metadata-aware integration by modeling patient identity as a covariate, allowing the variational autoencoder (VAE) to capture and correct for technical and biological heterogeneity across individuals. Second, SCANVI was used in conjunction with CellTypist annotations to incorporate cell-type–specific priors into the integration process, promoting biology-aware alignment across datasets. This strategy enabled robust and interpretable comparisons across patient samples from different tumor sites.

### Proportion analysis

A proportion analysis was conducted using the scProportionTest^118^ package to compare the fractions of cells within different cell populations. This involved performing a permutation test to calculate a p-value for each cluster and obtaining a confidence interval through bootstrapping.

### Constructing gene regulatory networks

We used pySCENIC^41^ to construct gene regulatory networks. This involved employing the GRNboost2 method for network inference. We used the *cisTarget* function with the Human motif database v10 to enrich gene signatures and pruned based on cis-regulatory cues using default settings. AUC scores were used to assess regulon enrichment across single cells, and the regulon specificity score was used to compute differential regulon activity.

### Copy number profile and subclone inference

We used the SCEVAN^39^ to determine clonal structures from inferred copy-number alteration profiles. The multiSampleComparisonClonalCN pipeline was employed for intratumoral comparison among multiple samples, with T cells and myeloid cells considered as the reference cells. Additionally, significant CNV groups distinguishing metastatic and primary malignant cells were identified using the InferCNV^23^ algorithm, grouping genes by chromosomal order and proximity, followed by permutation testing (10,000 permutations, p < 0.05) within each dataset. The overlap between significant CNV groups in different datasets were assessed.

### Pathway analysis

To assess the pathway activities, we employed decoupler-py^119^ and retrieved the gene sets from the Molecular Signatures Database (MSigDB)^59^, PROGENy^58^ database, and CancerSEA^70^ databases.

For each single-cell, pathway activity was inferred using the *decouple* function with default parameters applied to gene sets that included weight information for each gene. In cases where gene sets did not have weight information, we employed the *aucell* function with default parameters, also within the decoupler-py^119^ package.

### Data visualization

We used essential Scanpy^110^ functions to generate UMAPs, box plots, heatmaps, dot plots, and violin plots. Proportional change was analyzed and visualized by using scProportionTest^118^ and Pertpy^120^.

For each condition under consideration, we generated pseudobulk profiles in accordance with the official vignette for pseudo-bulk functional analysis using the decoupler-py algorithm. The decoupler-py^119^ *plot_barplot* function was employed to illustrate the top absolute value activities for pathway activities in each condition. Additionally, we utilized the *plot_targets* function to compare primary and metastatic gene expression profiles, focusing on the target genes associated with each PROGENy^58^ pathway.

Visualization of the dimension reduction of cellular states for pathway activities at the cellular level was performed using the SCpubr^121^ *do_CellularStatesPlot*. Gene signatures sourced from the Molecular Signatures Database (MSigDB)^59^ were utilized to create these visual representations.

### Differential abundance testing

Graph-based differential abundance analysis was performed using the Milo^122^ framework in Pertpy^120^ to compare the cellular composition between primary and metastatic samples. The analysis followed the tutorial provided in the Pertpy package and utilized integrated datasets for each major cell type.

### Cell-cell communication analysis

We investigated cell-cell communication using the CellChat v2^71^ tool to infer, visualize, and analyze cell-cell communication networks from scRNA-seq data. The analysis was conducted following the recommended pipeline provided by the CellChat authors^71^.

Furthermore, we utilized the MultiNicheNet^90^ package to conduct cell interaction analysis, predicting ligand-receptor interactions between different cell types in both primary and metastatic tumor microenvironments. The analysis followed the guidelines outlined in the MultiNicheNet vignette. We visualized each group’s top twenty-five ligand-receptor interactions using the *make_circos_group_comparison* function.

### Survival analysis

Kaplan-Meier survival analysis was performed using publicly available RNA-Seq datasets in the KM plotter^123^, focusing specifically on ER+ tumors among 2575 patients. We assessed the collective high or low expression of the following genes: ARNT, BIRC3, EIF2AK1, EIF2AK2, FANCA, HOXC11, KIAA1549, MSH2, MSH6, and MYCN.

### Methodology for IntClust Classification

We utilized the Cluster Independent Annotation (CIA)^55^ tool to compute scores for each IntClust and to classify each cell accordingly. The top 200 differentially expressed genes (DEGs) for each IntClust, as identified in the METABRIC^54^ study, were utilized as input for the CIA tool. Specifically, we utilized the *CIA_classify* function to calculate scores based on the expression levels of the selected DEGs and assign each cell to the most probable IntClust.

## Supplementary information

Supplementary Information

Supplementary Data 1

Supplementary Data 2

Supplementary Data 1

Supplementary Data 1

## Data Availability

Raw single-cell RNA sequencing data for this study are available in the NCBI BioProject database under accession number PRJNA1140267. Processed single-cell RNA sequencing data and single-cell RNA seq objects can be found as follows: 10.5281/zenodo.13743373.

## Code availability

The authors declare that all scripts supporting the findings of this study are available from the corresponding author upon reasonable request.
