# Supplementary material for: Single-cell RNA sequencing reveals different cellular states in malignant cells and the tumor microenvironment in primary and metastatic ER-positive breast cancer
Source: NPJ Breast Cancer. 2025 Aug 26;11:95. doi: 10.1038/s41523-025-00808-w (PMC12381257; doi:10.1038/s41523-025-00808-w)
Supplement: Supplementary file 1 — Supplementary Information [file 41523_2025_808_MOESM1_ESM.pdf]

Supplementary Figures for the Manuscript:

Single-cell RNA sequencing reveals different cellular states in malignant cells and the tumor microenvironment in primary and metastatic ER-positive breast cancer

Furkan Ozmen[1†*], Tugba Y. Ozmen[1†], Aysegul Ors[2†], Mahnaz Janghorban[1], Matthew J. Rames[1], Xi Li[1], Aaron Reid Doe[2], Fariba Behbod[3], Gordon B. Mills[1], Hisham Mohammed[2]

1Division of Oncological Sciences Knight Cancer Institute, Oregon Health & Science University, Portland, Oregon 97201, USA
2Cancer Early Detection Advanced Research Center, Knight Cancer Institute, Oregon Health & Science University, Portland, Oregon 97201, USA,
3University of Kansas Medical Center, Kansas City, KS
†These authors contributed equally to this work.

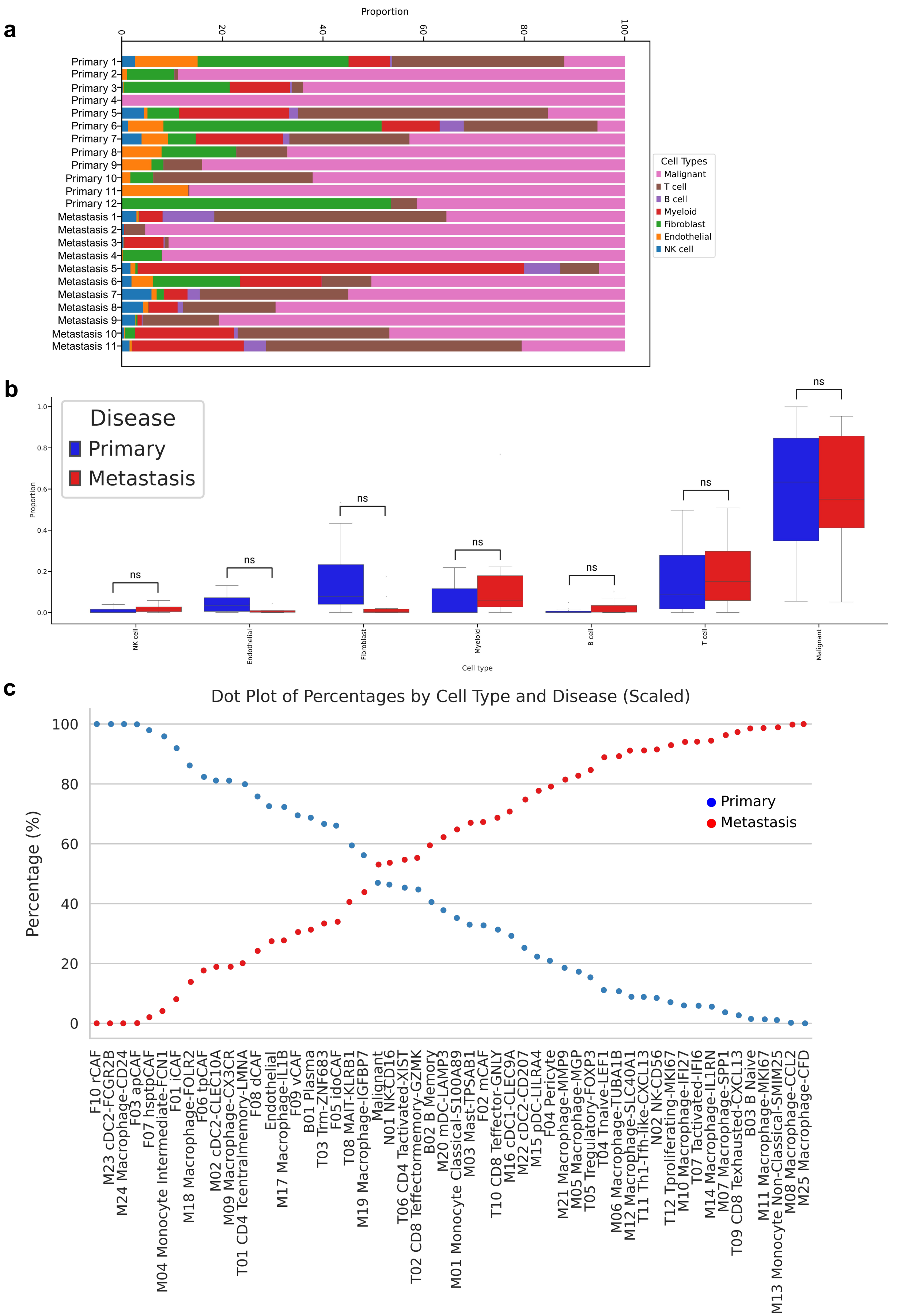

Supp. Fig. 1

a. Stacked bar plot showing the proportion of seven major cell types across individual samples.

b. Box plots illustrating changes in the proportions of seven major cell types between primary and metastatic breast cancer samples (permutation test, $p < 0.05$, log2FC > 2).

c. Dot plot illustrating the percentage of each cell subtype in primary versus metastatic tumors, highlighting the distribution and comparative abundance of each subtype across conditions.

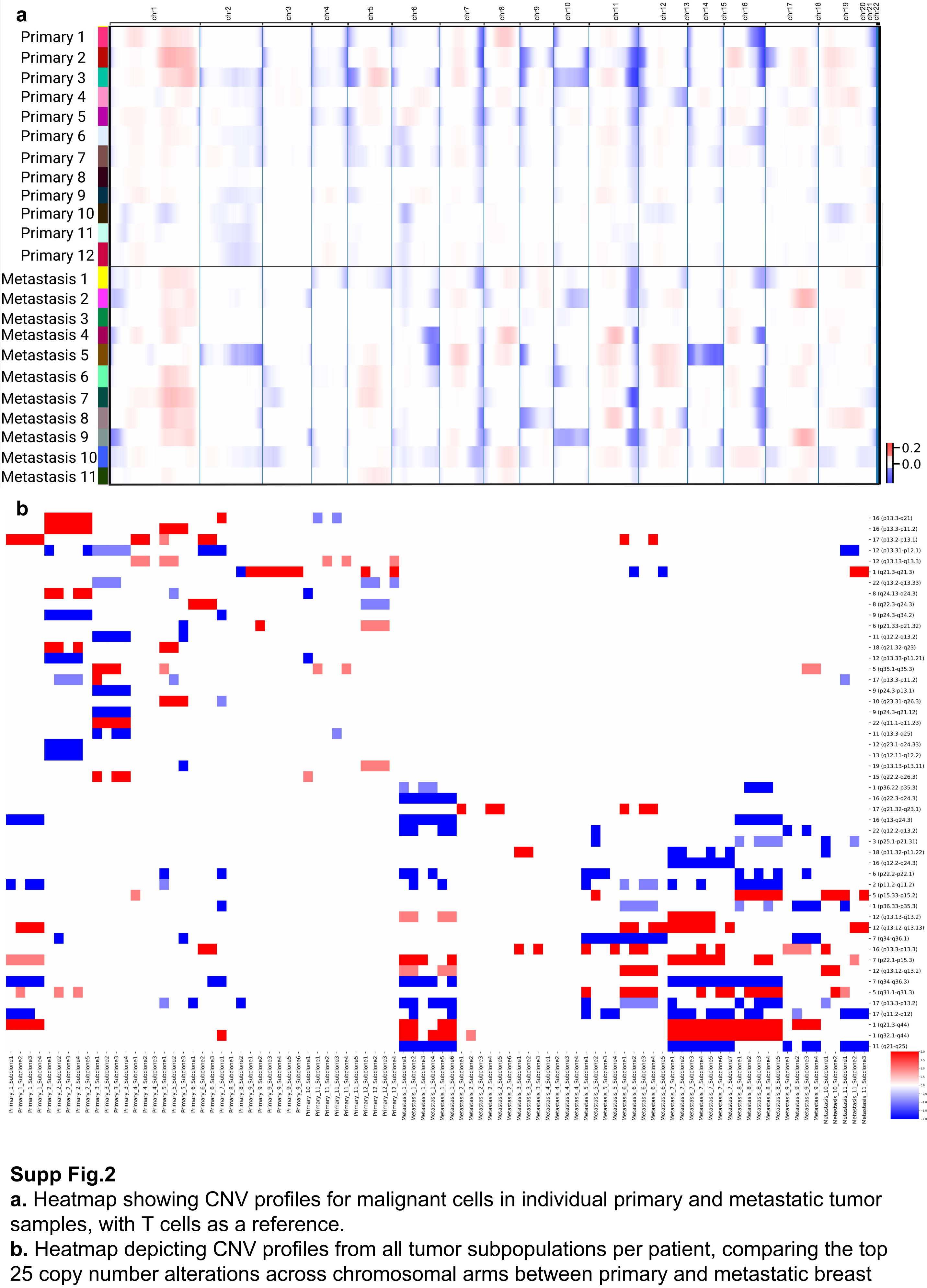

Supp Fig.2

a. Heatmap showing CNV profiles for malignant cells in individual primary and metastatic tumor samples, with T cells as a reference.

b. Heatmap depicting CNV profiles from all tumor subpopulations per patient, comparing the top 25 copy number alterations across chromosomal arms between primary and metastatic breast cancer subclones.

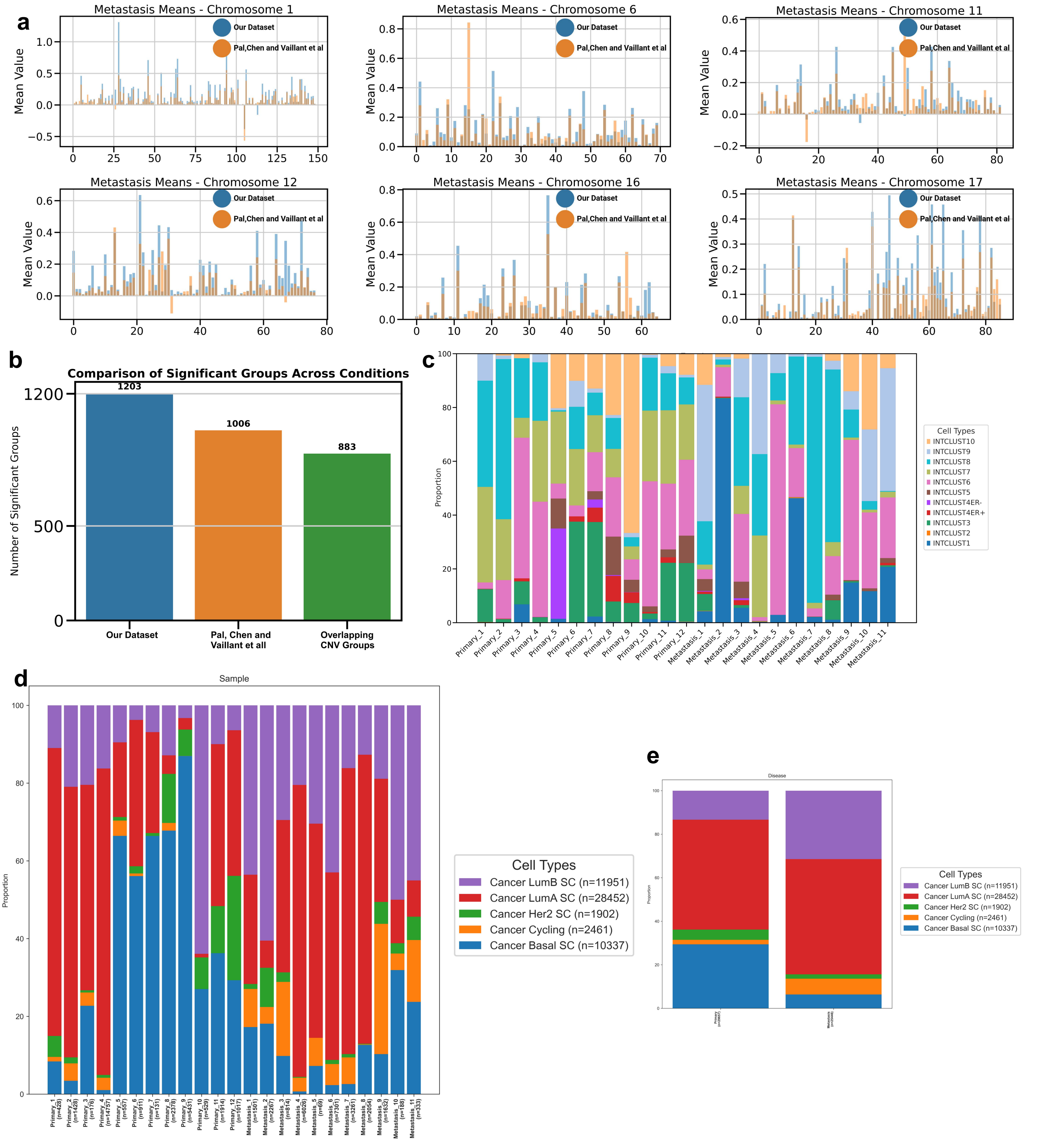

Supp. Fig. 3.

a. Bar plot comparing the mean CNV values of metastatic malignant cells between our dataset and publicly available ER+ metastatic samples from the Pal, Chen, and Vaillant et al. dataset[25].

b. Bar plot showing the number of significant CNV groups identified in our dataset and the Pal, Chen, and Vaillant et al. dataset[25], along with the number of overlapping significant groups.

c. Stacked bar plot showing the proportion of Integrative Clusters (IntClust) across individual samples, based on classification using the top 200 differentially expressed genes from the METABRIC study[53].

d. Stacked bar plot showing the PAM50 classification across samples in our dataset, based on the scRNA-seq dataset by Wu, Al-Eryani et al[56].

e. Stacked bar plot comparing the PAM50 subtypes in primary versus metastatic tumors in our dataset.

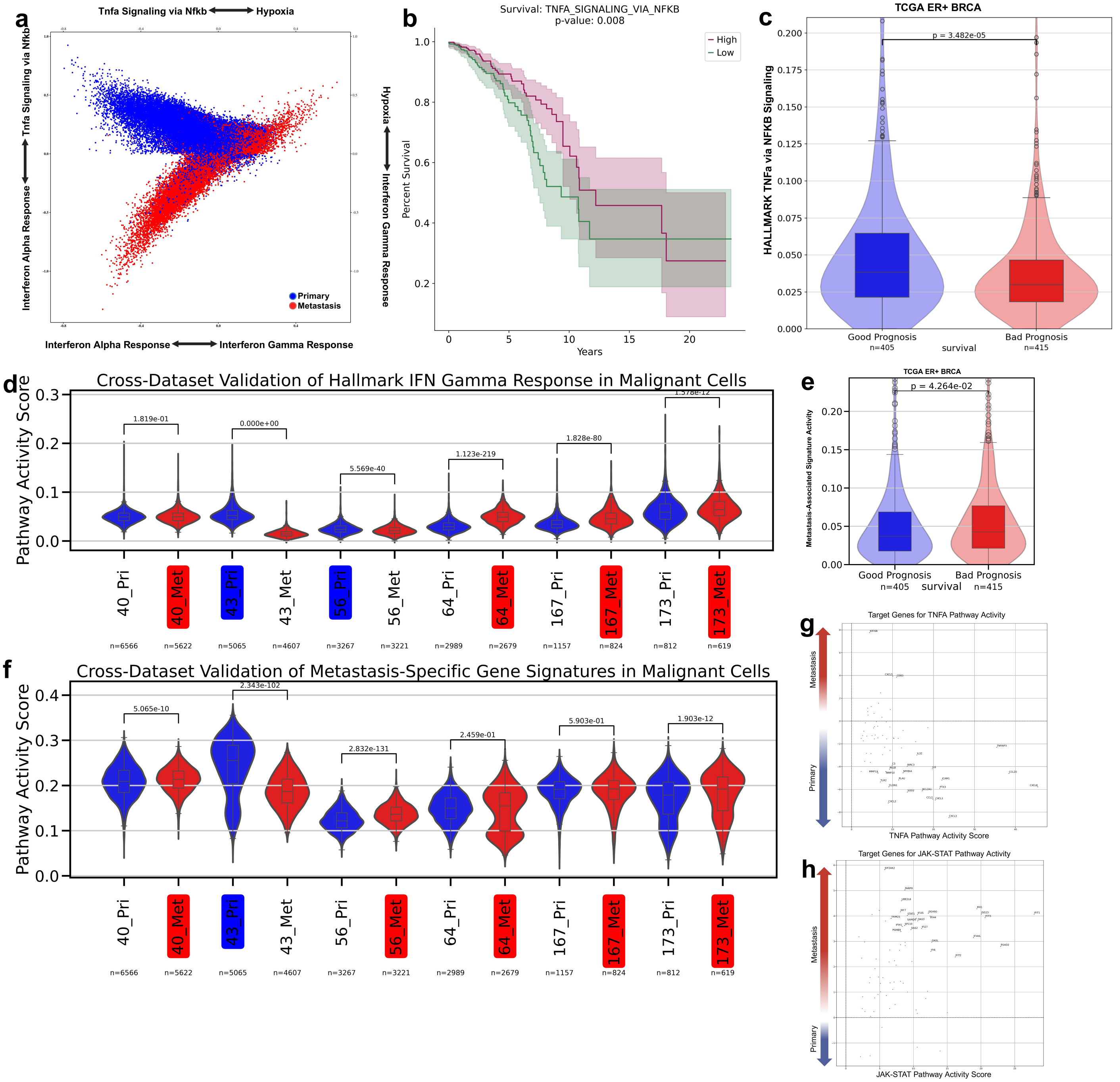

Supp. Fig. 4.

a. Cellular states plot showing Hallmark gene signature activity for Hypoxia, Interferon Gamma Response, Interferon Alpha Response, and TNFA Signaling via NFKB across metastatic status in malignant cells.

b. Kaplan-Meier plot of overall survival in ER+ breast cancer patients from the TCGA BRCA dataset[29], based on high vs. low TNF-α signaling via NF-κB.

c. Violin plot showing TNF-α signaling levels across prognosis groups (based on median overall survival) in ER+ patients (TCGA BRCA[29]).

d. Violin plot showing Hallmark IFN-gamma response activity in malignant cells from paired primary and metastatic samples of six ER+ breast cancer patients (Pal, Chen, and Vaillant et al.[25]).

e. Violin plot showing the metastatic gene signature expression (derived from our differentially expressed genes) in TCGA BRCA[29] dataset, stratified by prognosis.

f. Violin plot showing the same metastatic gene signature expression in malignant cells from the Pal, Chen, and Vaillant et al.[25] dataset.

g. Plot of the top 25 genes regulated by TNFA signaling, based on PROGENY pathway activity, in primary vs. metastatic malignant cells.

h. Plot of the top 25 genes regulated by JAK-STAT signaling, based on PROGENY pathway activity, in primary vs. metastatic malignant cells.

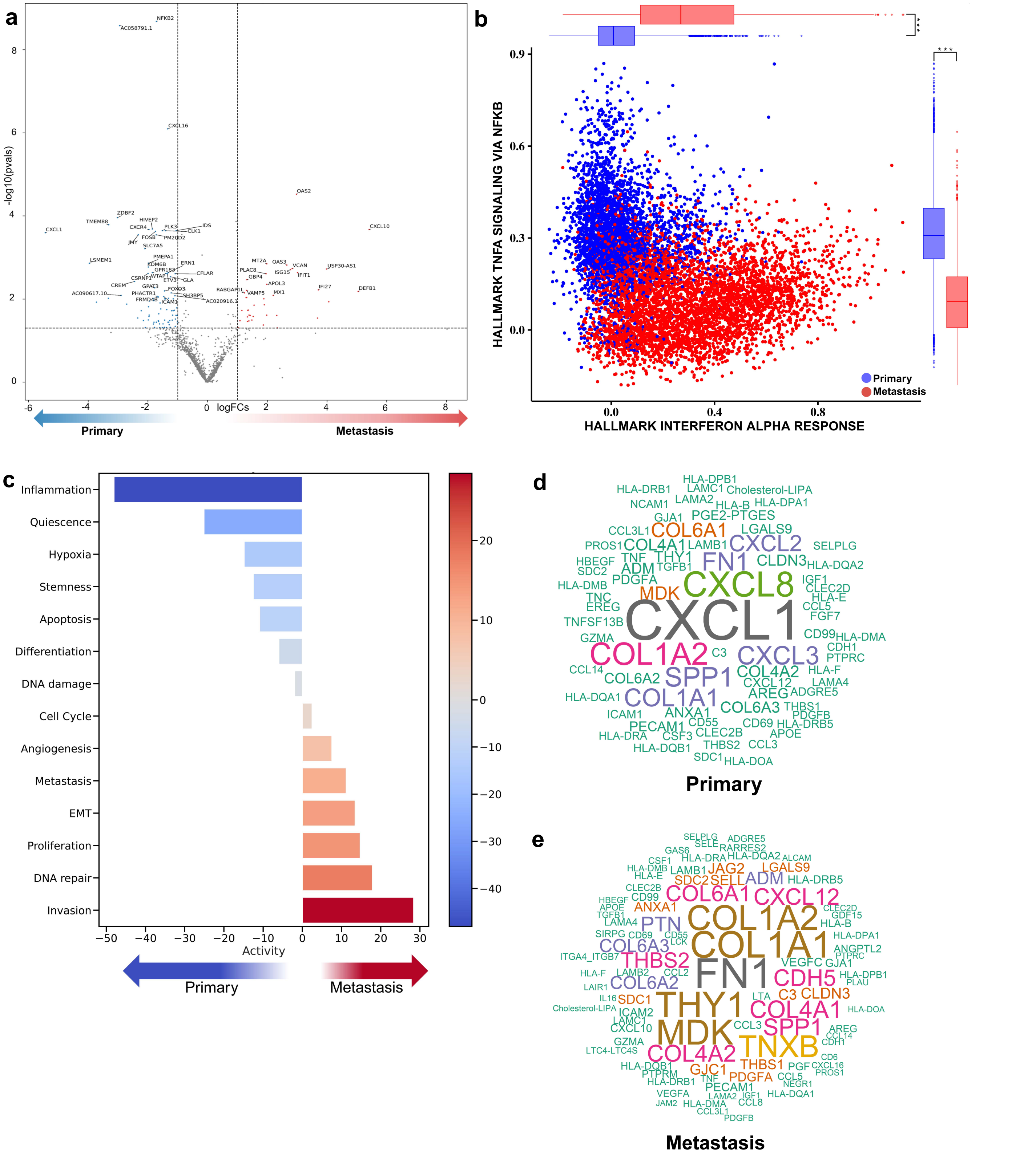

Supp. Fig. 5.

a. Volcano plot of differentially expressed genes in myeloid cells comparing primary vs. metastatic tumors.

b. Cellular states plot showing Hallmark Interferon Alpha Response vs. TNFA Signaling via NFKB in myeloid populations.

c. CANCERSEA gene signature pathway activity scores across metastatic status in myeloid cells.

d. Word cloud of enriched ligands in the primary tumor microenvironment compared to metastatic tumors.

e. Word cloud of enriched ligands in the metastatic tumor microenvironment compared to primary tumors.

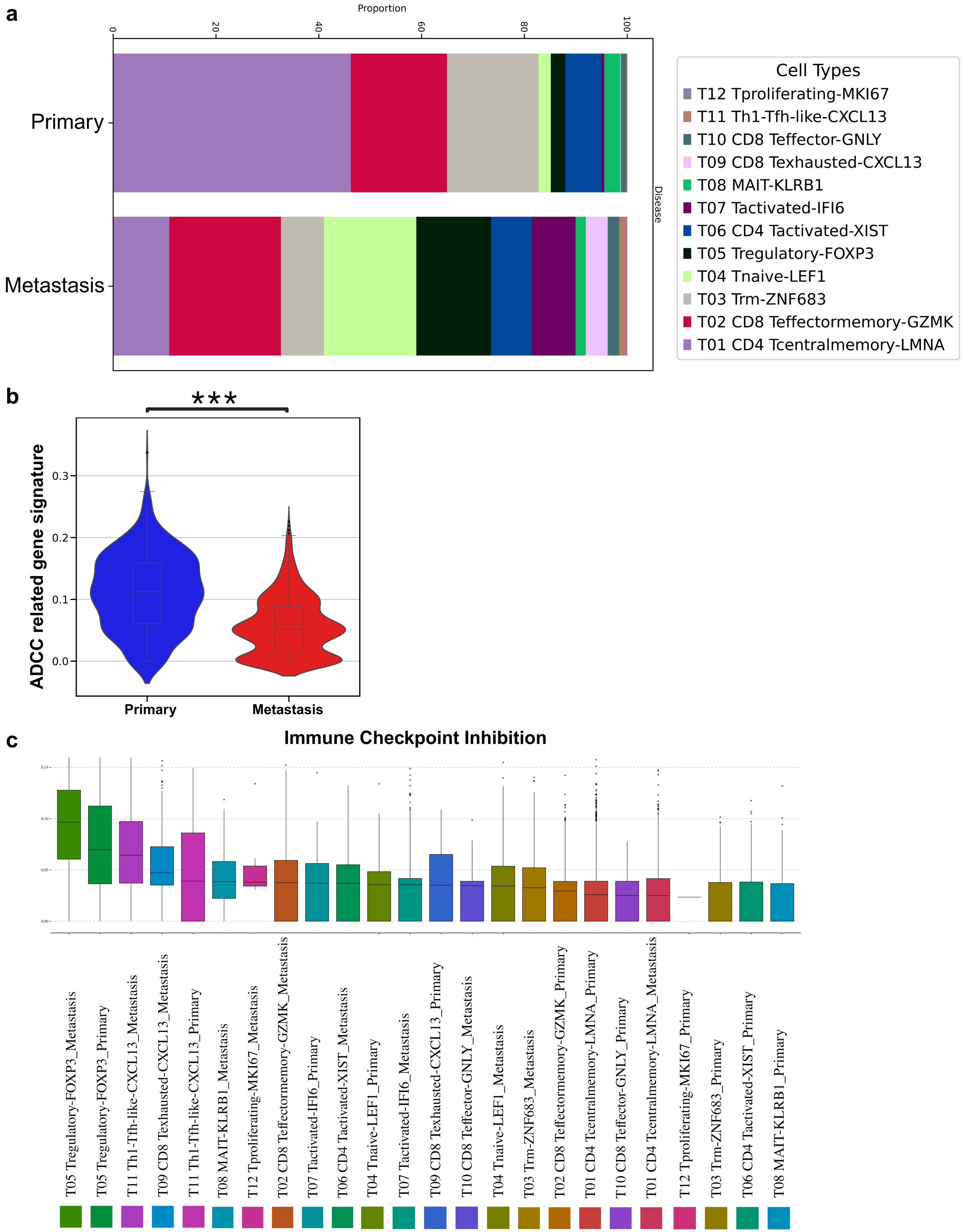

Supp. Fig. 6.

a. Stacked bar plot showing the proportion of T cell subtypes across metastatic status.

b. Comparison of antibody-dependent cellular cytotoxicity gene signature in NK cells from primary vs. metastatic tumors (***$p < 0.001$).

c. Comparison of immune checkpoint inhibition levels across different cell types between primary and metastatic tumors.

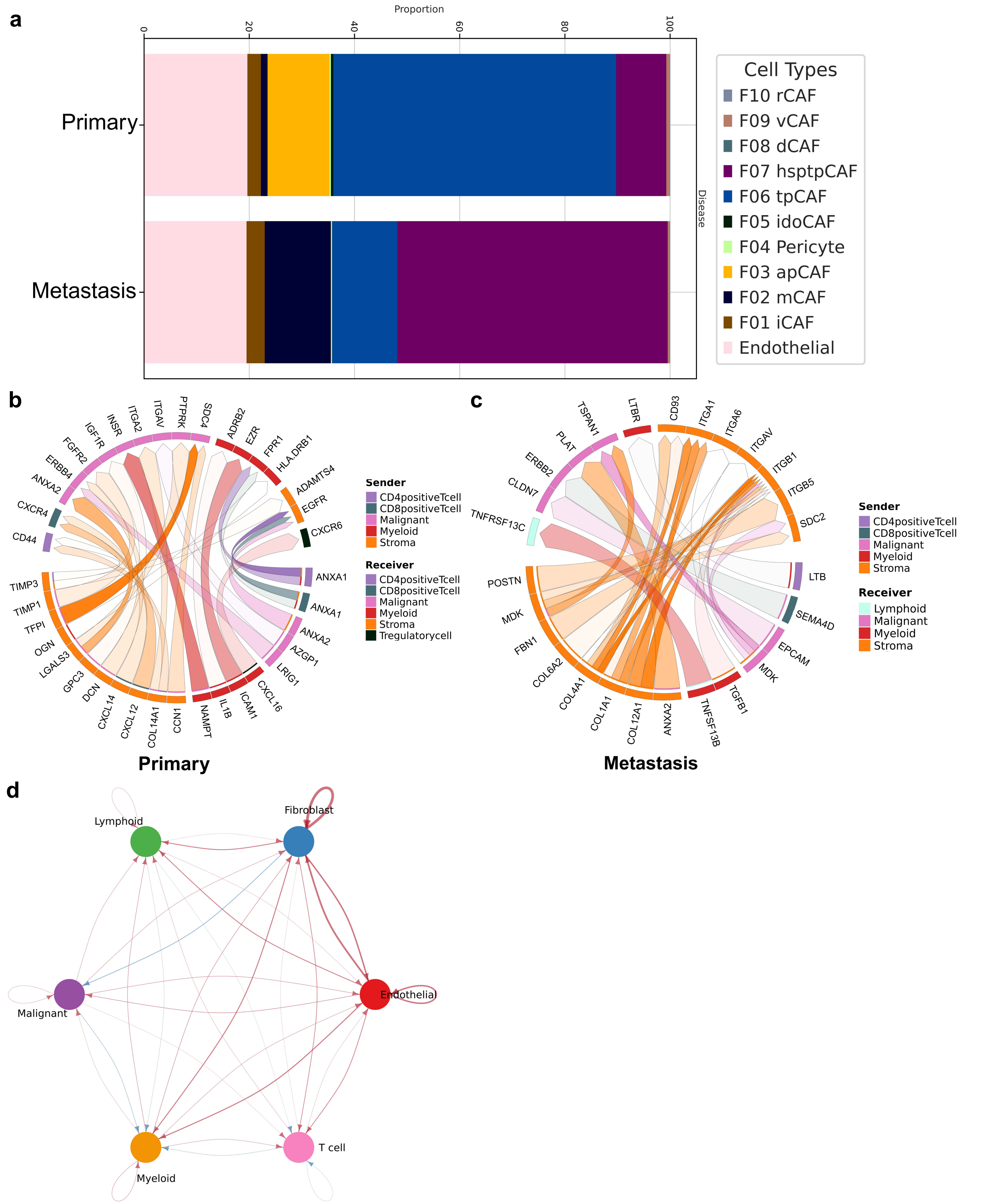

Supp. Fig. 7.

a. Stacked bar plot showing the proportion of stromal cell subtypes across metastatic status.

b. Top 25 ligand-receptor interactions between cell types in the primary tumor microenvironment, identified via MultiNicheNet.

c. Top 25 ligand-receptor interactions in the metastatic tumor microenvironment.

d. Comparative circle plot of differential cell-cell communication between primary and metastatic tumors, where red edges indicate increased interaction in metastatic tumors, and blue edges indicate increased interaction in primary tumors.

Overview of Supplementary Data

Supplementary Data 1. Clinical information related to samples in the study
This Excel file contains patient-specific clinical metadata, including diagnosis, sample type, site of origin (primary vs. metastasis), and other relevant clinical annotations used in the analysis.

Supplementary Data 2. Subclone information of malignant cells for individual samples
This Excel file includes the inferred subclonal structures and assignments for malignant cells across all tumor samples. It details subclone proportions, cluster identifiers, and sample-level distributions.

Supplementary Data 3. Cell type proportions and pathway activity comparisons
This Excel file provides a comprehensive summary of various comparisons between primary and metastatic breast cancer samples. It includes the proportions and counts of major and minor cell types, differentially expressed markers, and distributions of CNV and ITHGEX scores. The file also reports the frequencies of IntClust subtypes, regulon activity scores, and pathway activity analyses performed using PROGENy, Hallmark, and CancerSEA databases. It also includes site-specific differentially expressed genes identified in malignant cells across six metastatic locations (adrenal gland, bone, liver, lymphatic tissue, soft tissue, and thoracic cavity), capturing location-specific transcriptional programs. Gene signature scores used in the analysis are also provided.

Supplementary Data 4. CNV alteration heatmaps for individual samples
This Excel file contains sample-based visualizations of copy number variation (CNV) heatmaps across all tumor samples analyzed. Each heatmap is annotated with sample ID and segmented CNV patterns derived from single-cell profiles.
